# Molecular basis for the diversification of lincosamide biosynthesis by pyridoxal phosphate-dependent enzymes

**DOI:** 10.1038/s41557-024-01687-7

**Published:** 2024-12-06

**Authors:** Takahiro Mori, Yoshitaka Moriwaki, Kosuke Sakurada, Shuang Lyu, Stanislav Kadlcik, Jiri Janata, Aninda Mazumdar, Marketa Koberska, Tohru Terada, Zdenek Kamenik, Ikuro Abe

**Affiliations:** 1 Graduate School of Pharmaceutical Sciences, The University of Tokyo, Tokyo, Japan; 2 Collaborative Research Institute for Innovative Microbiology, The University of Tokyo, Tokyo, Japan; 3 PRESTO, Japan Science and Technology Agency, Saitama, Japan; 4 FOREST, Japan Science and Technology Agency, Saitama, Japan; 5 Graduate School of Agricultural and Life Sciences, The University of Tokyo, Tokyo, Japan; 6 Medical Research Laboratory, Institute of Science Tokyo, Tokyo, Japan; 7 Institute of Microbiology, Czech Academy of Sciences, Prague, Czech Republic

**Keywords:** Enzyme mechanisms, X-ray crystallography

## Abstract

The biosynthesis of the lincosamide antibiotics lincomycin A and celesticetin involves the pyridoxal-5′-phosphate (PLP)-dependent enzymes LmbF and CcbF, which are responsible for bifurcation of the biosynthetic pathways. Despite recognizing the same *S*-glycosyl-l-cysteine structure of the substrates, LmbF catalyses thiol formation through β-elimination, whereas CcbF produces *S*-acetaldehyde through decarboxylation-coupled oxidative deamination. The structural basis for the diversification mechanism remains largely unexplored. Here we conduct structure–function analyses of LmbF and CcbF. X-ray crystal structures, docking and molecular dynamics simulations reveal that active-site aromatic residues play important roles in controlling the substrate binding mode and the reaction outcome. Furthermore, the reaction selectivity and oxygen-utilization of LmbF and CcbF were rationally engineered through structure- and calculation-based mutagenesis. Thus, the catalytic function of CcbF was switched to that of LmbF, and, remarkably, both LmbF and CcbF variants gained the oxidative-amidation activity to produce an unnatural *S*-acetamide derivative of lincosamide.

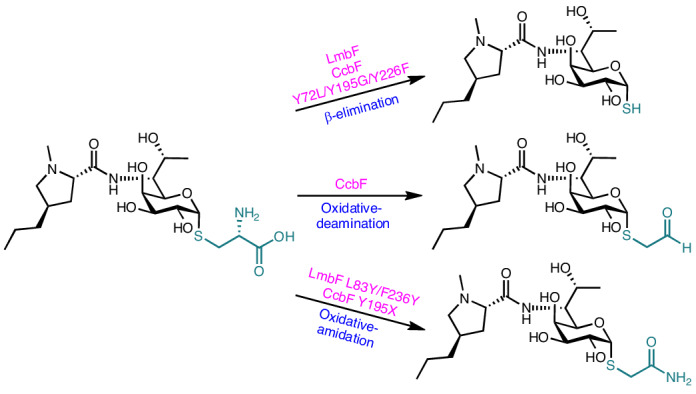

## Abstract (web summary)

The reaction mechanisms of the pyridoxal phosphate (PLP)-dependent enzymes LmbF and CcbF—involved in the biosynthesis of the antibiotic lincosamide—are characterized here. Structure–function analyses reveal structural features responsible for *S*-alkyl moiety diversification in lincosamides. Using structure- and calculation-based enzyme engineering, the selectivity profiles of these enzymes are designed to generate an unnatural lincosamide derivative.

## Main

**Fig. 1:**
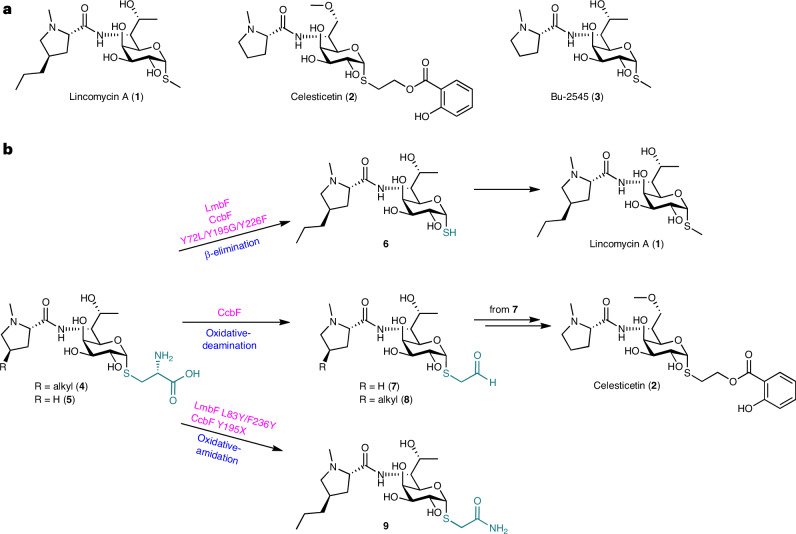
Biosynthesis of lincosamide antibiotics. **a**, Structures of lincosamide antibiotics. **b**, Enzyme reactions of LmbF, CcbF and LmbF/CcbF variants (LmbF L83Y/F236Y, CcbF Y195X and CcbF Y72L/Y195G/Y226F; X is Gly, Ala, Ser, Cys, Met, Asn, Gln or His). LmbF wild type and the CcbF Y72L/Y195G/Y226F variant catalyse the β-elimination reaction, and the CcbF wild type catalyses the oxidative-deamination reaction. LmbF L83Y/F236Y and CcbF Y195X variants catalyse the oxidative-amidation reaction.

Lincosamides, including lincomycin A (**1**)^1,2^, celesticetin (**2**)^3^ and Bu-2545 (**3**)^4^, are antibacterial natural products generated by *Streptomyces* species. Lincomycin and its semisynthetic derivative, clindamycin, are antibiotics that are clinically used against Gram-positive bacteria^5–8^. These compounds inhibit the early stage of protein synthesis by binding to the peptidyltransferase domain of the 50S ribosomal subunit^9,10^. The structure of lincosamide is characterized by a thiooctose core connected to *N*-methyl-(4*R*)-propyl-l-proline (*N*-methyl-PPL; in **1**) or *N*-methyl-l-proline (*N*-methyl-Pro; in **2** and **3**) and a differentially derivatized *S*-alkyl moiety^11,12^ (Fig. 1a).

Previous structure–activity relationship studies of lincosamides have suggested that *S*-functionalization of the thiooctose core is important for the strength of antibacterial activity^7,13^. The attachment of salicylate to the thiooctose core via a two-carbon alcohol improved the antibacterial activity compared to *S*-methyl or *S*-ethanol groups. The structural diversity of the *S*-alkyl moiety is generated by the pyridoxal-5′-phosphate (PLP)-dependent enzymes LmbF and CcbF in the biosynthesis of **1** and **2** (refs. ^8,14–17^). Although LmbF and CcbF share 40%/55% amino-acid identity/similarity, these enzymes accept the *S*-glycosyl-l-cysteines **4** and **5**, sharing the same l-cysteine-conjugated thiooctose core structure, as substrates, to catalyse different modification reactions (Fig. 1b). On the one hand, LmbF catalyses β-elimination with C–S bond cleavage of the l-cysteine moiety of **4** to generate the thiol **6**, which is then *S*-methylated by the methyltransferase LmbG to yield **1** (refs. ^15,16,18^). On the other hand, CcbF catalyses oxidative deamination coupled with decarboxylation of the cysteine moiety to yield the *S*-acetaldehyde **7**, which is subsequently reduced to form *S*-ethanol and converted into the salicylated **2** by downstream tailoring enzymes^8,15–17^. Interestingly, both LmbF and CcbF exhibit substrate promiscuity and accept **4**, **5** and the β-anomer of **5** as substrates, suggesting that these enzymes primarily recognize the l-cysteine moiety of the substrate^15,16^.

Mechanistically, in the LmbF reaction, a quinonoid intermediate with a carboxylate group is generated by deprotonation of the Cα-atom of the l-cysteine moiety, after formation of the external aldimine intermediate by the cysteine moiety binding to the PLP cofactor (Supplementary Fig. 1: quinonoid 1). The quinonoid intermediate then undergoes C–S bond cleavage to generate thiol **6** (ref. ^8^). In contrast, CcbF generates a different quinonoid species from the same external aldimine intermediate, through decarboxylation (Supplementary Fig. 1: quinonoid 2). Subsequently, in the presence of O_2_, a superoxide anion and semiquinone radical are thought to be formed via single-electron transfer from the decarboxylated quinonoid intermediate. The superoxide rebound to the semiquinone radical produces a hydroperoxyl intermediate, which is converted into the *S*-acetaldehyde **7** through H_2_O_2_ elimination and imine hydrolysis^8,19^. Thus, although the catalytic functions of LmbF and CcbF have been investigated in vivo and in vitro, the structural basis for the differences in reaction selectivity, including the detailed catalytic mechanism, remains to be elucidated.

In this Article we report structure–function analyses of LmbF and CcbF, catalysing the oxygen-independent β-elimination and the oxidative deamination, respectively. X-ray crystal structures, docking simulations with substrate **4** and molecular dynamics (MD) simulations of the enzymes demonstrated that the active-site tryptophan and tyrosine residues play crucial roles in controlling the binding mode of the substrate and the reaction outcome. Furthermore, site-directed mutagenesis revealed the molecular basis for the oxygen-utilization of the enzyme reactions, enabling structure-based rational engineering of the reaction selectivities of LmbF and CcbF. Thus, CcbF was functionally converted into LmbF, and, remarkably, both LmbF and CcbF variants gained the decarboxylation-coupled oxidative-amidation activity to produce an unnatural *S*-acetamide derivative of lincosamide.

## Results

### Structural analyses of LmbF and CcbF

**Fig. 2:**
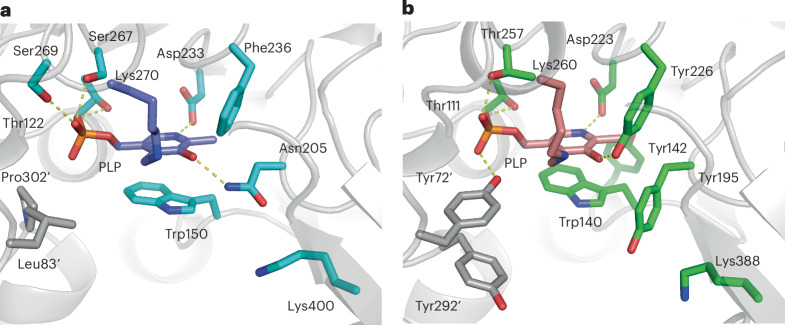
Active-site architectures of LmbF and CcbF. **a**,**b**, The active sites of the crystal structures of LmbF (PDB 8KDL; **a**) and CcbF in complex with PLP (PDB 8KDK; **b**). The active-site residues located near PLP are shown and labelled. Grey residues represent those from another monomer. Yellow dashes represent hydrogen-bond interactions. PyMol was used for figure preparation.

**Extended Data Fig. 1:**
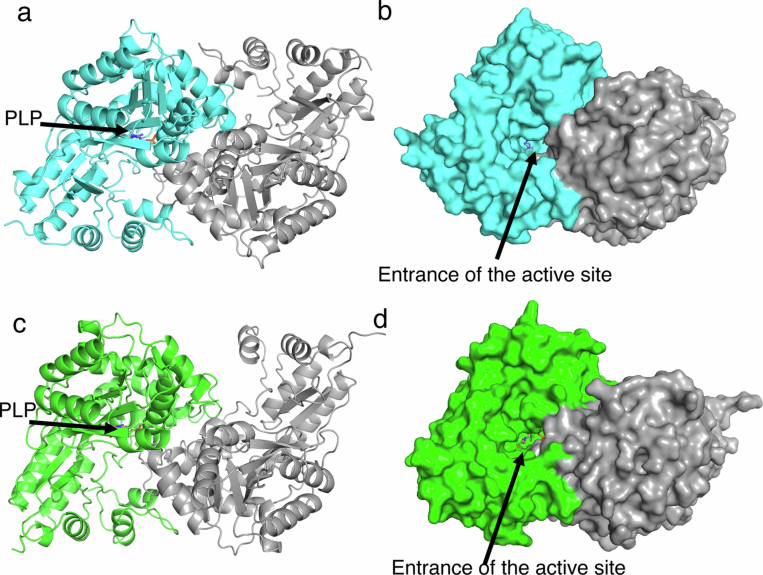
Overall structures of LmbF and CcbF. The structures of (**a**,**b**) LmbF and (**c**,**d**) CcbF.

To understand the molecular basis for the differences between LmbF and CcbF, we solved the X-ray crystal structures of LmbF and CcbF with PLP at resolutions of 1.7 Å and 1.8 Å, respectively. The overall structures of LmbF and CcbF possess type I folds^20^ and are highly homologous to each other (root-mean-squared deviation (r.m.s.d.) of 1.6 Å for the Cα-atoms of 386 amino acids; Extended Data Fig. 1). In the active sites of LmbF and CcbF, the PLP is covalently bound to the catalytic Lys270 and Lys260 residues, respectively, to form internal aldimines (Fig. 2). In the LmbF active site, the N1 and C3-hydroxy groups of PLP interact with Asp233 and Asn205, respectively, and the phosphate group of PLP hydrogen-bonds with Thr122, Ser267 and Ser269. Moreover, Trp150 interacts with the pyridine ring of PLP by *π*–*π* interactions. Similar hydrogen-bond network and *π*–*π* interactions are also observed in the active site of CcbF. Although the residues lining the active-site cavity are in comparable positions, Asn205, Phe236, Leu83′ and Pro302′ in LmbF are uniquely substituted with Tyr195, Tyr226, Tyr72′ and Tyr292′ in CcbF, respectively. These differences are thought to be important for the selectivity of the enzyme reactions (Fig. 2).

### MD simulations of LmbF and CcbF

**Fig. 3:**
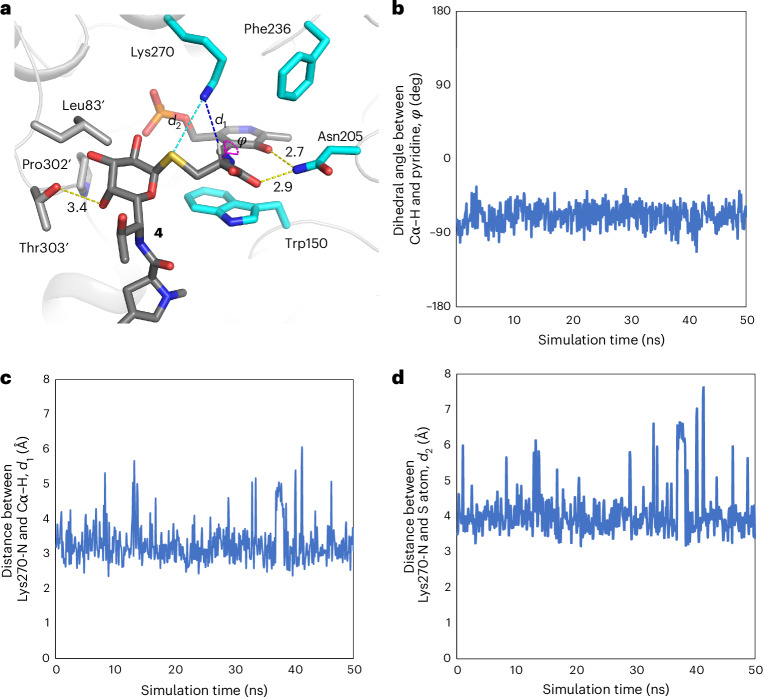
Docking simulation and MD simulation of LmbF with an external aldimine intermediate. **a**, Representative binding mode of the substrate in the active site of LmbF (PDB 8KDL). **b**, Time evolution of the dihedral angle between the Cα–H bond and the pyridine ring. **c**, Time evolution of the distance between the Cα–H atom and the amine atom of Lys270. **d**, Time evolution of the distance between the S atom of **4** and the amine atom of Lys270. These results suggest that the Cα–H bond is aligned parallel to the *p* orbitals of the conjugated *π*-system of PLP. PyMol was used for figure preparation. Source data

The Cα–X bond to be cleaved is thought to be positioned perpendicular to the pyridine ring of PLP, because the resulting negative charge at Cα is stabilized through resonance with the conjugated *π*-system of PLP^21–23^. To understand the structural basis for the different reaction selectivities between LmbF and CcbF, we performed docking and MD simulations based on the crystal structures. Docking of the external aldimine intermediate of **4** into the active site of LmbF and 50-ns simulations showed hydrogen-bond interactions involving the carboxylate group of the cysteine moiety of **4**, the C3-hydroxy group of PLP, and Asn205. The dihedral *ϕ* angle between the Cα–H bond and pyridine ring of PLP is stably maintained around −70° to −90°, indicating that the Cα–H bond is aligned parallel to the *p* orbitals of the conjugated *π*-system (Fig. 3a,b). The dihedral angle of N–Cα–C–S is −65°, and the octose moiety of **4** is within a pocket consisting of Leu83′, Pro302′, Thr303′ and PLP. As a result, the ε-amino group of Lys270 remains within ~2.5–3.5 Å of the Cα H atom (Fig. 3c), and the distance between the ε-amino group of Lys270 and the S atom of **4** is less than 3.5 Å in the calculation, suggesting that Lys270 probably acts as an acid–base catalyst to facilitate the C–S bond cleavage (Fig. 3d).

**Fig. 4:**
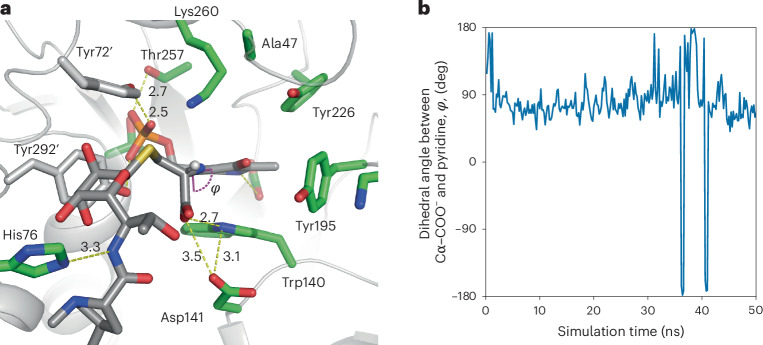
Docking simulation and MD simulation of CcbF with an external aldimine intermediate. **a**, Representative binding mode of the substrate in the active site of CcbF (PDBID, 8KDK). **b**, Time evolution of the dihedral angle between the Cα–COO^−^ bond and the pyridine ring. These results suggested that indicating that the Cα–COO^−^ bond is aligned parallel to the *p* orbitals of the conjugated π-system of PLP. PyMol was used for figure preparation. Source data

**Extended Data Fig. 2:**
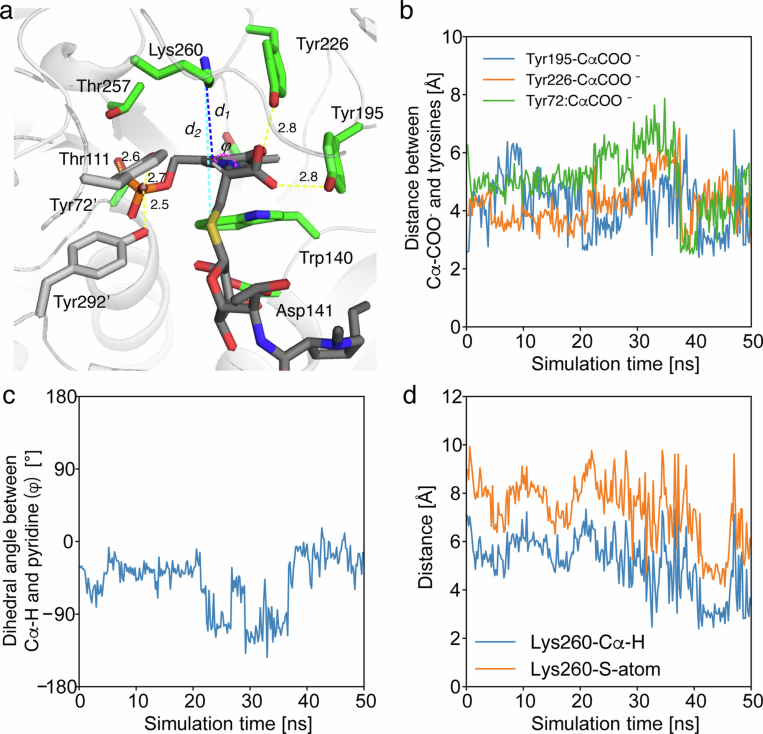
MD simulation of CcbF with external aldimine intermediate in a similar conformation to that in LmbF. (**a**) Representative conformation of the external aldimine intermediate in CcbF, in a similar conformation to that in LmbF. The side chain of the catalytic Lys260 is located between Tyr226 and Thr257 due to the steric hindrance between the side chain of Tyr72’ and the carboxylate group of **4**. Furthermore, the sulfur atom of **4** is located on the opposite side of the PLP from the catalytic Lys260, due to steric hindrance with Tyr72’ and Tyr292’. Yellow lines show hydrogen bond interactions. The blue and cyan lines indicate the distances from the amine atom of Lys260 to the Cα-H and the sulfur atom of **4**, respectively. (**b**) Time evolution of the distances between the carboxylate group and the active site Tyr72’ (green), Tyr195 (blue), and Tyr226 (orange). (**c**) Time evolution of the dihedral angle between the Cα-H bond and the pyridine ring. (**d**) Time evolution of the distances between the sulfur atom of **4** and the amine atom of Lys260 (orange) and the Cα-H and the amine atom of Lys260 (blue). Source data

In CcbF, His76 interacts with the amide of the substrate, and Trp140 and Asp141 form a hydrogen-bond network with the carboxylate of the cysteine moiety, maintaining an ~90° dihedral *ψ* angle between the Cα–COO^−^ bond and the pyridine ring of PLP (Fig. 4). The PPL moiety of **4** is flexible and located at the entrance of the active site (Fig. 4a), which is consistent with the substrate tolerance of CcbF. These differences in the binding modes of the cysteine moiety would allow CcbF to catalyse decarboxylation as the first step in the enzyme reaction. In contrast, MD simulations of CcbF with the external aldimine intermediate in a similar conformation to that in LmbF showed unstable binding of the carboxylate (Extended Data Fig. 2). The interactions between the carboxylate of **4** and the active-site residues are altered among Tyr72′, Tyr195 and Tyr226 by rotation of the Cα–N bond in the active site, and the dihedral *ϕ* angle changes between 0° to −90° during the simulation (Extended Data Fig. 2c). Furthermore, the ε-amino group of Lys260 is not accessible to the Cα−H atom and the S atom of **4**, due to the steric hindrance among the side chains of Lys260, Tyr72′ and Tyr292′ and the octose moiety of **4** (Extended Data Fig. 2a,d and Supplementary Fig. 2). As a result, the LmbF-like conformation is thought to be a non-reactive binding mode in CcbF. These in silico analyses suggest that the active-site Trp140 and the Tyr72′, Tyr195, Tyr226 and Tyr292′ residues in CcbF play important roles in substrate binding and selectivity in the first step of the enzyme reaction. Notably, although the active-site loop dynamics are important for the general catalytic mechanism of aromatic l-amino-acid decarboxylases (AADC), large conformational changes in the loop region were not observed in the active site of LmbF and CcbF^24–27^.

**Extended Data Fig. 3:**
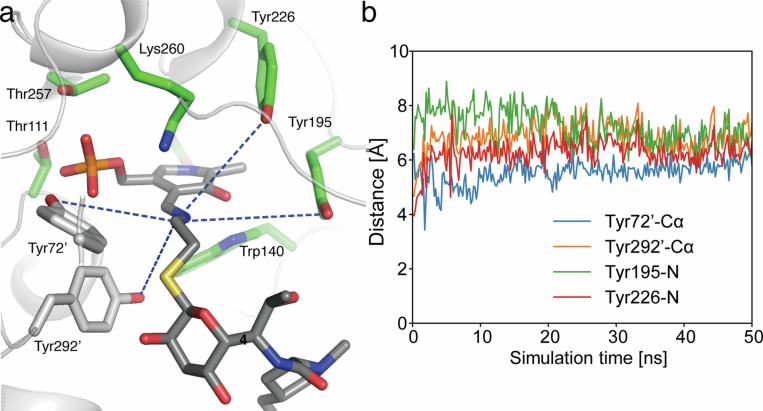
MD simulation of CcbF with quinonoid intermediate. (**a**) Representative conformation of the quinonoid intermediate in CcbF. Blue lines indicate the distance between the quinonoid intermediate and active site tyrosines. (**b**) Time evolution of the distance between the quinonoid intermediate and the active site Tyr195 (green), Tyr226 (red), Tyr72’ (blue), and Tyr292’ (orange) residues. Source data

In the CcbF reaction, following decarboxylation, the quinonoid intermediate reacts with molecular oxygen to yield a semiquinone and superoxide anion (Supplementary Fig. 1). Plant and insect AADCs utilize histidine and tyrosine residues to protonate the quinonoid intermediate to generate decarboxylated products^24–27^. Although the tyrosine residue in plant AADCs and the histidine residue in insect AADCs are conserved, these residues are substituted with other amino acids such as phenylalanine and asparagine, respectively, in amino-acid aldehyde synthases (AAASs; Supplementary Fig. 3). Accordingly, the quinonoid intermediate is not protonated, and instead it reacts with molecular oxygen to catalyse oxidative deamination in AAASs. In CcbF, the histidine residue is replaced with Trp140. Docking of the quinonoid intermediate into the active site of CcbF and MD simulations suggest that, despite the presence of four tyrosine residues, Tyr195, Tyr226, Tyr72′ and Tyr292′, the distances from the substrate’s Cα–N bond are greater than 4.5 Å. Accordingly, these tyrosine residues do not interact with Trp140 or other amino acids (Extended Data Fig. 3). These observations clearly suggest that the Cα of the substrate is not protonated by the active-site residues, as in the cases of AASs.

### Site-directed mutagenesis of CcbF

**Fig. 5:**
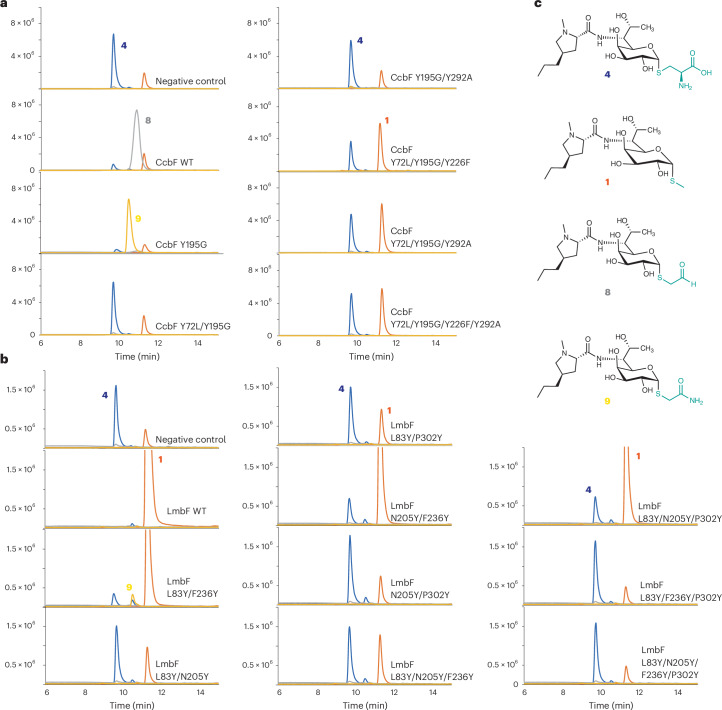
Mutagenesis studies of LmbF and CcbF. **a**, LC–MS profiles of the enzyme reaction products of LmbF wild type and its variants. **b**, LC–MS profiles of the enzyme reaction products of CcbF wild type and its variants. Blue, orange, grey, and yellow plots represent extracted ion chromatography (EIC) = 480 (for **4**), EIC = 407 (for **1**), EIC = 435 (for **8**), and EIC = 450 (for **9**) respectively. **c**, Structures of the substrate (**4**) and the products (**1**, **8**, **9**). Source data

**Extended Data Fig. 4:**
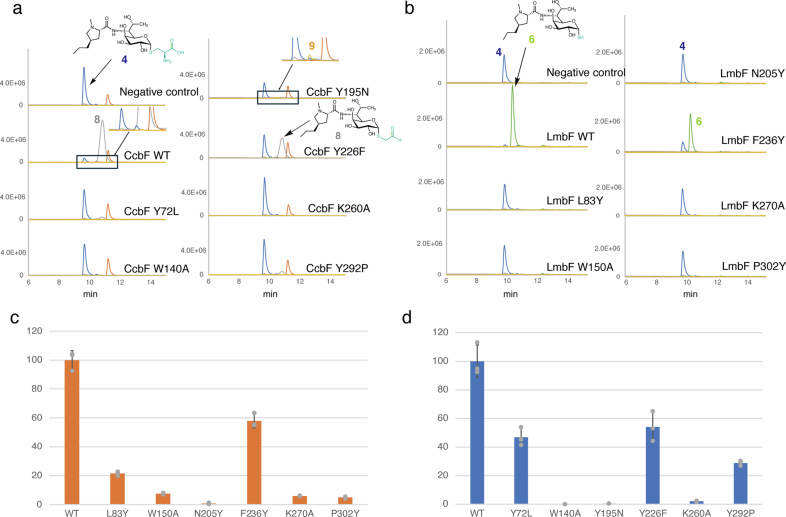
Mutagenesis analysis of LmbF and CcbF single variants. (**a**) LC-MS profiles of the enzyme reaction products of CcbF wild type and its variants with LmbG (2 h reaction). (**b**) LC-MS profiles of the enzyme reaction products of LmbF wild type and its variants (5 min reaction). Blue, green, orange, gray, and yellow charts represent EIC = 480 (for **4**), EIC = 393 (for **6**), EIC = 407 (for **1**), EIC = 435 (for **8**), and EIC = 450 (for **9**), respectively. (**c**) Comparison of the relative activities of **6** production by LmbF wild type and its variants for 10 min. (**d**) Comparison of the productivities of **8** by CcbF wild type and its variants for 14 h. All reactions were performed in triplicate. Data are presented as mean values, and error bars indicate standard deviations (SD). Source data

**Extended Data Fig. 5:**
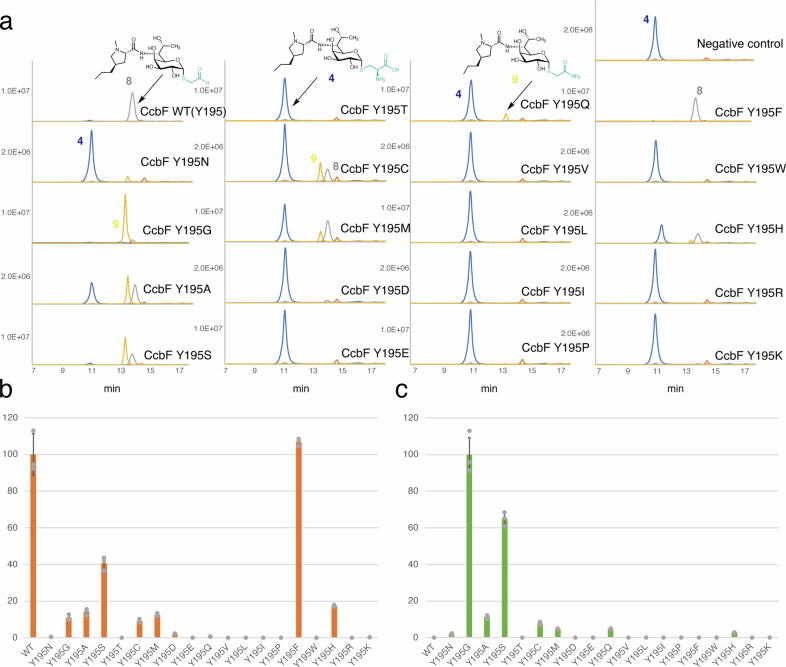
Site-saturation mutagenesis study of Tyr195 in CcbF. (**a**) LC-MS profiles of the enzyme reaction products of CcbF wild type and its Y195X variants. Blue, gray, and yellow charts represent EIC = 480 (for **4**), EIC = 435 (for **8**), and EIC = 450 (for **9**), respectively. (**b**) Comparison of the productivities of **8** by CcbF wild type and its variants (production of wild type is defined as 100%). (**c**) Comparison of the productivities of **9** by CcbF wild type and its variants (production of Y195G is defined as 100%). All reactions were performed in triplicate. Data are presented as mean values, and error bars indicate standard deviations (SD). Source data

**Extended Data Fig. 6:**
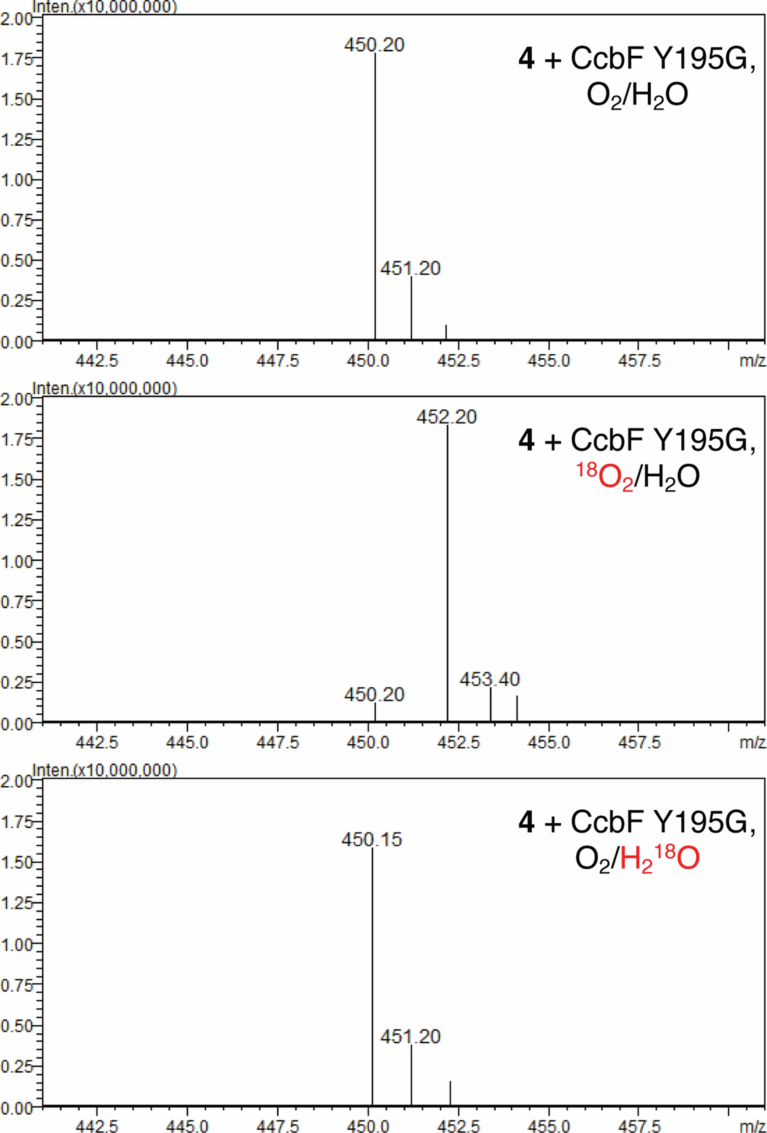
Enzyme reaction of CcbF Y195G in the presence of ^18^O_2_ or H_2_^18^O. The enzyme reactions containing ^16^O_2_ and H_2_^16^O, ^18^O_2_ and H_2_^16^O, and ^16^O_2_ and H_2_^18^O.

Site-directed mutagenesis was performed to investigate the importance of the active-site residues in CcbF for the reaction selectivity. The key residues, Tyr72′, Trp140, Tyr195, Tyr226, Lys260 and Tyr292′, were substituted with the corresponding residues in LmbF (Leu83′, Asn205, Phe236 and Pro302′) or alanine. As a result, the Y72L, Y226F and Y292P variants retained the **8**-producing activity (47%, 54% and 29% of wild type for 14 h), but W140A, Y195N and K260A almost lost this activity (Extended Data Fig. 4a,c). Interestingly, although the CcbF Y195N variant dramatically reduced the oxidative-deamination activity, it generated the distinct product **9** with *m*/*z* = 450 (Extended Data Fig. 4a). Further site-saturated mutagenesis of residue 195 revealed that the Y195G variant substantially increases the production of **9** (Fig. 5a and Extended Data Fig. 5). The structure of **9** was determined to be the *S*-acetamide derivative of lincosamide, which has never been reported before (Supplementary Table 1 and Supplementary Fig. 4). Oxygen isotopic labelling experiments indicated that molecular oxygen serves as the oxygen source in this reaction (Extended Data Fig. 6).

**Extended Data Fig. 7:**
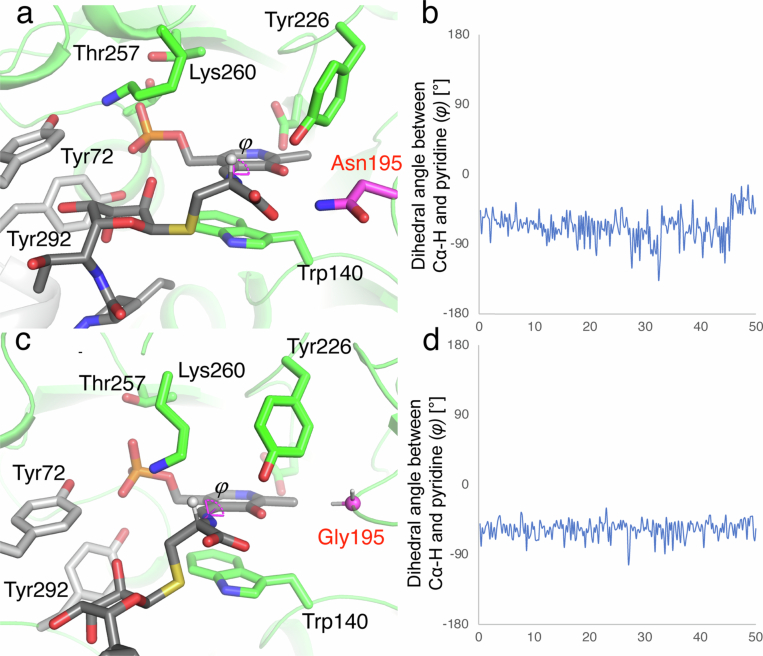
Comparison of CcbF Y195N and Y195G with substrate 4. (**a**) Representative conformation of the external aldimine intermediate in CcbF Y195N. (**b**) Time evolution of the dihedral angle between the Cα-H bond and the pyridine ring of CcbF Y195N. (**c**) Representative conformation of the external aldimine intermediate in CcbF Y195G. (**d**) Time evolution of the dihedral angle between the Cα-H bond and the pyridine ring of CcbF Y195G. Source data

Comparisons of the docking and MD simulations of the CcbF Y195N and Y195G models suggested that the carboxylate of the cysteine moiety in Y195G is more stably located in the space created by the large-to-small substitution of Tyr195 with Gly, and the Cα–H bond adopts the position perpendicular to PLP (Extended Data Fig. 7). These observations, together with the result that the Y195F variant maintained its oxidative-deamination activity, suggested that the steric hindrance at position 195 is critical for controlling the reaction selectivity (deprotonation versus decarboxylation) of the first step of the enzyme reaction, primarily through steric effects. However, due to steric hindrances from the octose moiety, Tyr72′ and Tyr292′, the S atom remains distant from Lys260 during the simulation, inhibiting effective protonation for β-elimination (Extended Data Fig. 7a,c). To further optimize the activity, multiple-site variants based on CcbF Y195G were constructed. Interestingly, the CcbF Y72L/Y195G/Y226F and Y72L/Y195G/Y292A variants completely switched the reaction selectivity to β-elimination, thus converting the catalytic function of CcbF to that of LmbF (Fig. 5a), and highlighting the roles of Tyr72′, Tyr226 and Tyr292′ in governing the reaction selectivity of the second enzyme reaction step following the deprotonation from Cα–H. The octose moiety is accommodated in the space created by the large-to-small substitution of Tyr72′ or Tyr292′ to bind in a similar conformation to that in LmbF, which is consistent with the docking and MD simulation results of LmbF (Fig. 3).

### Site-directed mutagenesis of LmbF

Next, mutations at the Leu83′, Trp150, Asn205, Phe236, Lys270 and Pro302′ of LmbF were introduced to alter its reaction selectivity. The in vitro enzyme reactions of the LmbF L83Y, W150A, N205Y, F236Y, K270A and P302Y variants revealed that the β-elimination activity is abolished or dramatically decreased in most cases (less than 10% compared to wild type), except for the L83Y and F236Y variants, which showed 21% and 58% activities, respectively (Extended Data Fig. 4b,d). Although most of the multiple-site mutations at Leu83′, Asn205, Phe236 and Pro302′ of LmbF merely reduced or abolished the β-elimination activity, the LmbF L83Y/F236Y variant catalysed the decarboxylation-coupled oxidative amidation to produce **9**, as well as the β-elimination reaction (Fig. 5b). Further triple and quadruple variants, including the LmbF L83Y/N205Y/F236Y, L83Y/F236Y/P302Y and LmbF L83Y/N205Y/F236Y/P302Y variants, decreased the oxidative-amidation activity, indicating that the subtle conformational difference of the cysteine moiety is critical for the utilization of molecular oxygen by LmbF (Fig. 5b).

### Reaction selectivities of LmbF and CcbF

**Extended Data Fig. 8:**
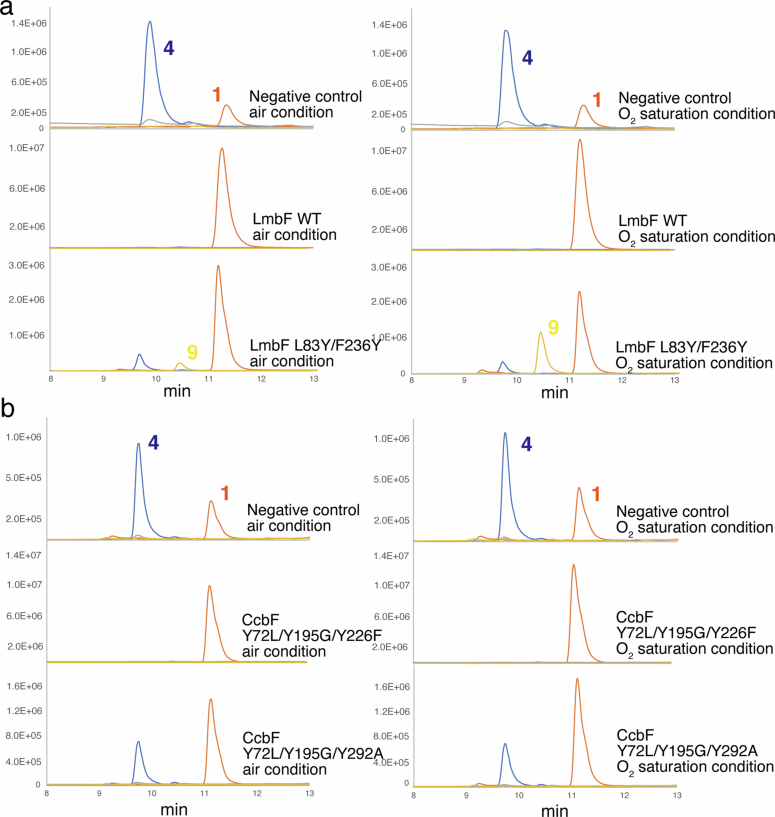
Enzyme reaction of LmbF and CcbF in saturated O_2_ condition. (**a**) LC-MS profiles of the enzyme reaction products of LmbF wild type and LmbF L82Y/F236Y with LmbG. (**b**) LC-MS profiles of the enzyme reaction products of CcbF wild type, CcbF Y72L/Y195G/Y226F, and CcbF Y72L/Y195G/Y292A. Blue, orange, and yellow charts represent EIC = 480 (for **4**), EIC = 407 (for **1**), and EIC = 450 (for **9**), respectively. Source data

The reaction of LmbF L83Y/F236Y under saturated oxygen conditions showed a fivefold increase in **9**-producing activity, suggesting that oxygen-molecule accessibility is one of the rate-limiting steps of the decarboxylation-coupled oxidative-amidation reaction (Extended Data Fig. 8). In contrast, LmbF wild type and the CcbF Y72L/Y195G/Y226F and Y72L/Y195G/Y292A mutants did not produce **9** even under saturated oxygen conditions. These observations also indicate that the oxygen-utilization is strictly controlled by Leu83′ and Phe236 in LmbF, but Tyr72′, Tyr226, and Tyr292′ in CcbF.

**Extended Data Fig. 9:**
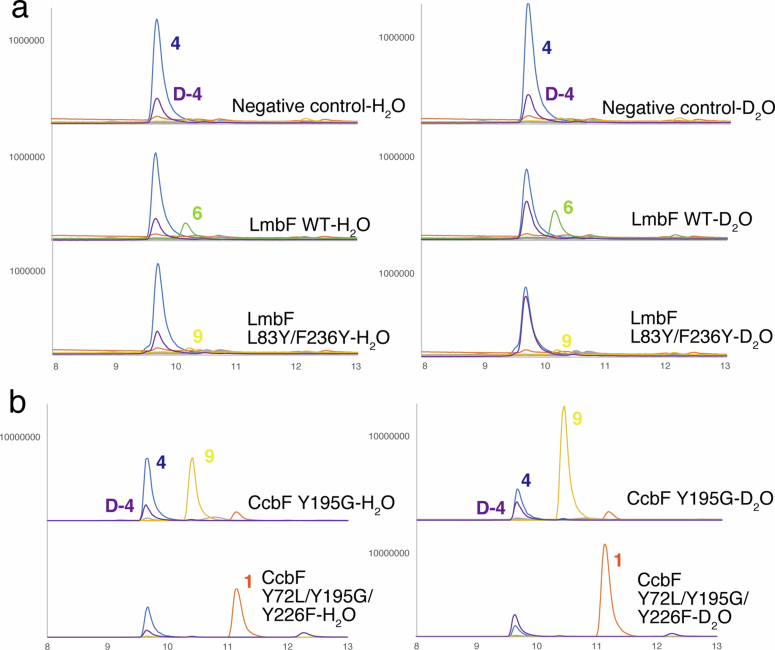
The enzyme reactions of1 LmbF and CcbF in the presence of D_2_O. (**a**) LC-MS profiles of the enzyme reaction products of LmbF wild type and its variants. (**b**) LC-MS profiles of the enzyme reaction products of CcbF variants with LmbG. Blue, purple, green, orange, gray, and yellow charts represent EIC = 480 (for **4**), EIC = 481 (for D-**4**), EIC = 393 (for **6**), EIC = 407 (for **1**), EIC = 435 (for **8**), and EIC = 450 (for **9**), respectively. Source data

Some PLP-dependent enzymes catalyse direct substrate release after quinonoid formation through protonation to quinonoid intermediates and subsequent nucleophilic attack by the catalytic lysine^28^. To further analyse the β-elimination and decarboxylation-coupled oxidative-amidation reactions, which are initiated by deprotonation from the Cα position, enzyme reactions of LmbF wild type and L83Y/F236Y, and CcbF Y195G and Y72L/Y195G/Y226F were conducted in the presence of D_2_O. The ratio of D-labelled **4** increased during the enzyme reactions, and the reaction efficiencies increased due to the kinetic isotopic effect on the protonation step (Extended Data Fig. 9). These results suggest that β-elimination and oxidative amidation compete with protonation of the quinonoid intermediate after Cα–H deprotonation.

## Discussion

PLP-dependent enzymes catalyse a wide range of reactions, including β- or γ-elimination/substitution, oxidative deamination, desaturation, hydroxylation, (3 + 2) annulation, Mannich reaction, oxidative amide-bond formation, decarboxylation, transamination, racemization, retro-aldol cleavage, aldol condensation and Claisen condensation^20,22,23,29–35^. Their catalytic versatility has attracted keen attention as a source of powerful biocatalysts^36–38^. LmbF and CcbF play key roles in the biosynthesis of lincosamide antibiotics, catalysing β-elimination and decarboxylation-coupled oxidative deamination, respectively^8,12,15–17^. Similar reactions to LmbF are observed in the cystathionine β- or γ-lyases^39,40^, the carbon-sulfoxide lyases Egt2 (ref. ^41^) and EgtE^42^, and the cysteine lyase domain in the biosynthesis of leinamycin^43^. In the cystathionine β-lyases^39,40^ and Egt2 (ref. ^41^), the carboxylate group of the amino acid is anchored by conserved arginine and partially conserved asparagine residues (Supplementary Fig. 5). In LmbF, the arginine residue is substituted with Lys400, but it does not interact with the cysteine moiety of **4**. Instead, the C3-hydroxy group of PLP and Asn205 interact with the carboxylate of **4**, indicating a slight difference in binding compared to other C–S β-lyases. In PLP-dependent decarboxylases^25,44,45^, conserved histidine residues interact with the carboxylates of substrates, placing the C–COO^−^ bond perpendicular to the pyridine ring (Supplementary Fig. 5). Although the histidine is substituted with Trp140 in CcbF, MD simulations and mutagenesis experiments suggest a similar function of Trp140 in positioning the carboxylate moiety in the active site.

Among PLP-dependent enzymes, those that utilize molecular oxygen are relatively rare^19,33,34,46–48^. Decarboxylation-coupled oxidative deamination is also observed in the paracatalytic reactions of fold-type I decarboxylase and aldehyde synthases^23^ (Supplementary Fig. 6). The key structural difference between the enzymes catalysing decarboxylation and decarboxylation-coupled oxidative deamination is the presence of the residues responsible for protonation of the quinonoid intermediate^19,25^. In the presence of proton donors like tyrosine or histidine, Cα-protonation of the quinonoid intermediate yields decarboxylated products, whereas the same quinonoid intermediate reacts with oxygen molecules in the absence of proton donor residues. Interestingly, although CcbF has four tyrosine residues in the active site, the Cα of the quinonoid intermediate is isolated from these tyrosines to prevent its protonation.

The CcbF and LmbF mutagenesis studies highlighted the pivotal roles of four active-site residues in the reaction selectivity by affecting the binding positions of the Cα-carboxylate and the C–S bond of the cysteine moiety. Tyr195 in CcbF is crucial for the selectivity of the first reaction step through steric effects with the Cα-carboxylate, and the Y195G variant favours decarboxylation-coupled oxidative amidation to produce **9**. Leu83′ and Phe236 in LmbF influence the selectivity between β-elimination and oxidative amidation. Because the L83Y and F236Y single variants fail to yield **9**, the interactions between the carboxylate of **4** and Tyr236 and the steric hindrance between the sugar moiety of **4** and Tyr83′ are both critical for utilizing molecular oxygen in the reaction. Furthermore, the CcbF Y72L/Y195G/Y226F and Y72L/Y195G/Y292A variants, containing the mutation of Tyr72′ (Leu83′ in LmbF), catalyse oxygen-independent β-elimination. These observations suggest that the Tyr residues at position 72 in CcbF and position 83 in LmbF play especially important roles in the selectivity of oxygen-independent β-elimination and oxygen-dependent decarboxylation-coupled oxidative amidation. However, because the CcbF Y72L variant still catalyses oxidative deamination, the oxygen molecule does not directly bind to the Tyr residues. In addition to the steric effect between the substrate and the tyrosine residues, the interactions between the lysine and tyrosine may also prevent the protonation of the C–S bond for β-elimination after formation of the external aldimine intermediate, thereby allowing oxygen uptake to react with the quinonoid intermediate in the active site.

**Fig. 6:**
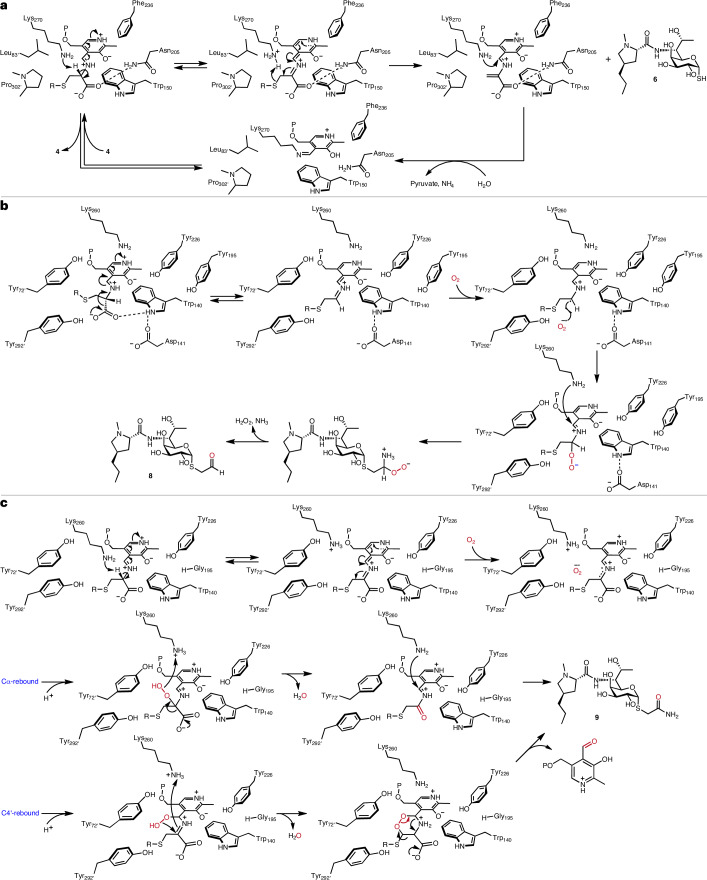
Proposed reaction mechanisms of LmbF and CcbF. **a**–**c**, Enzyme reaction mechanisms of β-elimination by LmbF (**a**), decarboxylation-coupled oxidative deamination by CcbF (**b**) and decarboxylation-coupled oxidative amidation by CcbF/LmbF variants (CcbF Y195G; **c**).

Based on the structural studies, the mechanisms for the **6** and **7** formation reactions by LmbF and CcbF are proposed as follows (Fig. 6). In the LmbF reaction, the amino group of the substrate’s cysteine moiety attacks the imine of the internal aldimine intermediate to form the external aldimine intermediate. Here, the catalytic Lys270 is positioned in proximity to both the Cα–H atom of the cysteine moiety and the S atom of the thiooctose. The Lys270 then abstracts a H atom from the Cα-position of the cysteine as a base catalyst, to afford the quinonoid intermediate. The subsequent C–S bond cleavage, supported by Lys270 as an acid catalyst, yields the thiol product. The CcbF variants catalysing β-elimination showed lower efficiency compared to LmbF, suggesting that the positioning of the C–S bond of the quinonoid intermediate and Lys260 is not suitable for the β-elimination reaction. Consequently, the protonation from Lys260 and the following nucleophilic attack to release the substrate compete with the β-elimination reaction.

The LmbF-like conformation is not stable in CcbF due to interactions between the carboxylate of **4** and Tyr72′, Tyr195 and Tyr226, as well as the steric hindrance among the octose and Tyr72′ and Tyr292′. Interactions of the carboxylate with Trp140 align the Cα–COO^−^ bond parallel to the *p* orbital of the pyridine ring, enabling decarboxylation to form the quinonoid intermediate. Afterwards, due to the absence of a proton donor residue, the quinonoid intermediate donates an electron to an oxygen molecule to generate the semiquinone radical and superoxide. The semiquinone radical and superoxide would then react to form a hydroperoxy intermediate, and the elimination of H_2_O_2_ and imine hydrolysis generate the aldehyde product.

Oxidative-amidation reactions to form carboxamides are also catalysed by Cap15 and PvdN in the biosynthesis of capuramycins and pyoverdines, respectively^33,34,49–51^. The Cap15 mechanism involves the abstraction of a Cα–H atom by a catalytic lysine to form a quinonoid intermediate^51^. Docking and MD simulations of the CcbF Y195G variant suggested a similar mechanism to Cap15, with a catalytic lysine abstracting the Cα proton for the formation of **9** (Fig. 6c and Supplementary Fig. 7). Single-electron transfer to an oxygen molecule is followed by peroxide radical rebound to the Cα or C4′ carbon. From the Cα-peroxyl intermediate, decarboxylation with the release of hydroxide and subsequent re-formation of the internal aldimine would generate *S*-acetamide derivative **9**. Alternatively, from the C4′-peroxyl intermediate, nucleophilic attack from the C4′ peroxide would produce a cyclized intermediate, followed by decarboxylation to yield **9**.

## Conclusions

The structure–function analyses of LmbF and CcbF revealed the intimate structural details underlying the diversification of the *S*-alkyl moiety of lincosamides, which is important for modulating the biological activities of the antibiotics. Furthermore, structure- and calculation-based enzyme engineering rationally altered the reaction selectivities of LmbF and CcbF. Remarkably, both LmbF and CcbF variants gained decarboxylation-coupled oxidative-amidation activity to produce an unnatural *S*-acetamide derivative of lincosamide. These findings provide insights into the catalytic mechanisms of the PLP-dependent enzymes and strategies towards expanding their catalytic repertoire to generate further structural diversity of antibiotics for drug discovery.

## Methods

### General

Oligonucleotide primers and DNA sequencing services were provided by Eurofins Genomics. Restriction enzymes and PrimeSTAR MAX DNA polymerase were purchased from Takara Bio Inc. Solvents and chemicals were purchased from Wako Chemicals, Merck KGaA and Hampton Research, unless noted otherwise. Polymerase chain reaction (PCR) was performed using a TaKaRa PCR Thermal Cycler Dice Gradient system (TaKaRa Bio). NMR spectra of compounds were recorded on Bruker AVANCE III HD 900 MHz and ECX-500 MHz (JEOL) spectrometers.

### Construction of plasmids for LmbF and CcbF expression

The *lmbF* gene was PCR-amplified from the chromosomal DNA of the lincomycin-producing-type strain *Streptomyces lincolnensis* ATCC 25466, using the following primers: forward CCGCATATGTCCGACTTAGCTGCCGTTGATGC and reverse CCGCTCGAG CCGGTACCGCCACTCGGCCGCGG. The *lmbF* gene was inserted into the pET42b vector (Novagen) via the NdeI and XhoI restriction sites. The resulting plasmid was used to produce C-terminally His_8_-tagged LmbF. The *ccbF* gene was PCR-amplified from chromosomal DNA of the celesticetin-producing-type strain *Streptomyces caelestis* ATCC 15084, using the following primers: forward CCGCATATG TCCGACTTAGCTGCCGTTGATGC and reverse CCGCTCGAGGCGGGGCTGCCAGGCGCGTGAGG. The *ccbF* gene was inserted into the pET42b vector (Novagen) via the NdeI and XhoI restriction sites. The resulting plasmid was used to produce C-terminally His_8_-tagged CcbF.

### Expression and purification of CcbF, LmbF and their variants

The pET42b plasmids for the expression of CcbF, LmbF and their variants were transformed into *Escherichia coli* BLR(DE3) harbouring the pGro7 plasmid. The resulting strains were cultured in LB medium supplemented with 34 mg l^−1^ chloramphenicol and 50 mg l^−1^ kanamycin sodium at 37 °C, with shaking at 160 r.p.m. When the optical density at 600 nm (OD_600_) reached 0.6, the cell cultures were cooled on ice for 30 min, then isopropyl β-d-thiogalactopyranoside (IPTG; 0.3 mM) was added to induce the target protein expression and the cultures were maintained at 16 °C, 160 r.p.m. After 18 h of post-induction incubation, cells were collected by centrifugation at 5,500*g* for 10 min and suspended in lysis buffer containing 20 mM Tris-HCl (pH 8.0), 100 mM NaCl, 5 mM imidazole and 10% glycerol. The cell suspension was sonicated for 5 min on ice. After the cell debris was removed by centrifugation at 20,000*g* for 30 min, the supernatant was mixed with 1 ml of TALON resin and loaded onto a gravity flow column. Unbound proteins were removed with 100 ml of lysis buffer containing 20 mM imidazole, then the His-tagged protein was eluted with lysis buffer containing 300 mM imidazole. For the in vitro assay, the eluted enzymes were concentrated after buffer exchange to 20 mM Tris-HCl (pH 8.0), 100 mM NaCl, 5 mM imidazole, 0.5 mM EDTA, 1 mM dithiothreitol (DTT) and 10% glycerol, using a 30-kDa Amicon Ultra-15 filtration unit (Millipore).

For crystallization, the His-tag-purified enzymes were applied to a 6-ml Resource Q anion exchange chromatography column (4 °C, Cytiva) and a HiLoad 16/60 Superdex 200 prepacked gel filtration column (4 °C, GE Healthcare), and eluted with a solution containing 20 mM HEPES (pH 8.0), 100 mM NaCl and 1 mM DTT. The resulting eluate was concentrated to 7 mg ml^−1^ using an Amicon Ultra-4 (molecular weight cutoff, 30 kDa) filter at 4 °C. The purity of the proteins was monitored by sodium dodecyl sulfate polyacrylamide gel electrophoresis (SDS–PAGE; Supplementary Fig. 12), and the protein concentrations were determined with a SimpliNano microvolume spectrophotometer. The stability of the variants was tested by incubating the enzymes at 37 °C for 1 h. The aggregated proteins were separated by centrifugation, and the proteins in the supernatants and precipitates were analysed by SDS–PAGE (Supplementary Fig. 13). Notably, the stabilities of the LmbF and CcbF variants were comparable to those of the wild types.

### In vitro assays of LmbF, CcbF and their variants

The standard enzymatic reaction of LmbF with substrate **4** was performed in a 12.5-μl volume containing 100 mM potassium phosphate (KPi, pH 8.0), 40 μM substrate **4**, 0.5 mM EDTA, 0.5 mM tris(2-carboxyethyl)phosphine (TCEP), 0.5 mM PLP and 0.2 μM enzyme, and incubated for 5 min at 37 °C. For the in vitro analysis of the multiple mutants of LmbF, 12.5-μl reaction mixtures containing 100 mM KPi (pH 8.0), 40 μM substrate **4**, 0.5 mM EDTA, 0.5 mM DTT, 0.3 mM *S*-adenosyl-methionine (SAM), 0.1 mM PLP, 4 μM wild type or LmbF variants, and 10 μM of LmbG to convert unstable product **6** to stable lincomycin A (**1**), were incubated for 60 min at 37 °C. The standard enzymatic reaction of CcbF and its variants with substrate **4** was performed in a 12.5-μl volume containing 100 mM KPi (pH 8.0), 40 μM substrate **4**, 0.5 mM EDTA, 0.5 mM DTT, 0.1 mM PLP, 0.1 mM SAM, 20 μM wild type or mutant CcbF, and 20 μM of LmbG to convert the unstable product **6** to stable lincomycin A (**1**), and incubated overnight at 37 °C. Liquid chromatography mass spectrometry (LC–MS) samples were injected into a Shimadzu Labsolution LCMS 8045 system. A COSMOSIL 5C18-MS-II packed column (2.0 × 50 mm, 5 μm; Nacalai Tesque) was used for separation. Gradient elution was performed with solvent A (1 mM ammonium formate, pH 9.0) and solvent B (CH_3_CN), with a flow rate of 0.2 ml min^−1^ (min, % of B: 0 min, 10%; 3 min, 10%; 12 min, 100%; 15 min, 100%), followed by 5 min of equilibration with 10% solvent B before the next analysis.

For the O_2_ saturation reaction, a premixed solution containing 100 mM KPi, 0.5 mM EDTA, 0.5 mM DTT, 0.3 mM SAM and 0.1 mM PLP was saturated with O_2_ by bubbling O_2_ gas for 30 min before reactions, then 20 μM wild type or mutant CcbF was incubated in 25-μl reaction mixtures containing 100 mM KPi, 0.5 mM EDTA, 0.5 mM DTT, 0.3 mM SAM, 0.1 mM PLP, 40 μM substrate **4**, 10 μM LmbG, prepared from the O_2_-saturated solution for 120 min at 37 °C under an O_2_ atmosphere. The reactions were quenched by adding 100 μl of methanol. Reactions without O_2_ bubbling were performed as negative controls. The samples were analysed with the same method as that used for the standard reaction.

### Labelling experiments

For the ^18^O_2_ labelling experiment, 25-μl reactions were performed in 500-μl tubes containing 100 mM KPi (pH 8.0), 40 μM substrate **4**, 0.5 mM EDTA, 0.5 mM DTT, 0.5 mM PLP and 0.2 μM CcbF Y195G, combined under anaerobic conditions. Afterward, ^18^O_2_ gas (98%) or air was injected into the vial. For the H_2_^18^O labelling experiment, 10 μl of H_2_^18^O (97%) was added for the preparation of 25-μl reaction solutions (final concentration of H_2_^18^O of 38.8%). The enzyme reactions were performed overnight at 20 °C and quenched by adding 100 μl of methanol. The samples were stored at −20 °C until LC–MS analysis to decrease the exchange rate with solvents. The products were analysed with the same method as that for the standard reaction products.

For the deuterium labelling experiments of LmbF and its variant, 15.7 μl of D_2_O (99.8%) was added for the preparation of 25-μl reaction mixtures (final concentration of D_2_O of 62.7%) containing 100 mM KPi, 0.5 mM EDTA, 0.5 mM DTT, 0.1 mM PLP, 40 μM substrate **4**, and 0.1 nM wild type or 2 μM mutant LmbF. After incubation for 5 min at 37 °C, the reactions were quenched by adding 100 μl of methanol. For the deuterium labelling experiments of CcbF and its variants, 15.7 μl of D_2_O (99.8%) was added for the preparation of 25-μl reaction mixtures (final concentration of D_2_O of 62.7%) containing 100 mM KPi, 0.5 mM EDTA, 0.5 mM DTT, 0.3 mM SAM, 0.1 mM PLP, 40 μM substrate **4**, 10 μM LmbG and 4 μM wild type or mutant CcbF. After incubation for 120 min at 37 °C, the reactions were quenched by adding 100 μl of methanol. Reactions in 100% H_2_O were performed as negative controls. The samples were analysed with the same method as that for the standard reaction.

### Production and purification of the natural thiooctose substrate of LmbF and CcbF (4)

The seed culture of the *S. lincolnensis* Δ*lmbIH* strain^17^ was prepared by inoculating spores into 50 ml of Yeast Extract-Malt Extract (YEME) medium in 500-ml flat-bottom boiling flasks, which were incubated at 28 °C for 30 h, 180 r.p.m. A 2-ml portion of the seed culture was then inoculated into 40 ml of avermectin medium (AVM) medium in 500-ml flat-bottom boiling flasks, and incubated at 28 °C for 120 h. The supernatants from 30 flat-bottom boiling flasks were used in the next steps. The cells were centrifuged at 4,000*g* at 4 °C for 10 min, and the supernatant was stored at −20 °C. The compound of interest was extracted in two steps. First, a glass column containing Amberlite XAD-4 was used, and the amount of the sorbent was ~5 cm in diameter and 10 cm in height. Methanol (MeOH) followed by water was used to equilibrate the column before loading the supernatant, which was adjusted to pH 9–10 with ammonium hydroxide. The sorbent was then washed with 1 mM ammonium formate (pH 9). Absorbed compounds were subsequently eluted with a methanol–water solvent system: from methanol–water 10:90 (vol/vol) up to 80:20 (vol/vol) in steps of 10% and a 100-ml volume of each solvent. Fractions including the compound of interest were collected, pre-concentrated and adjusted to pH 2–3 with formic acid. Next, MCX 35 cc (6 g) (Waters) cartridges were used. The cartridge was conditioned and equilibrated with MeOH followed by 2% formic acid, then the eluate collected from the previous extraction was applied to the cartridge, which was washed with 2% formic acid and MeOH. Thereafter, 200 ml of MeOH with 5% of an aqueous solution of ammonium hydroxide (29%) was loaded to elute the compound of interest. For further purification of the natural substrate, a two-step preparative scheme was employed. The first step was performed with a Triart Prep C18-S column (20 × 250 mm, 5 μm; YMC) using a two-component mobile phase: A, 0.1% formic acid and B, methanol. Elution was performed at a flow rate of 3.5 ml min^−1^ with a linear gradient (min, % of B: 0 min, 5%; 27.5 min, 63%; 28 min, 100%; 43 min, 100%), followed by 15 min of equilibration with 5% solvent B. The second step was performed on an Xterra Prep RP18 column (7.8 × 150 mm, 5 μm; Waters) using a two-component mobile phase: A, 1 mM ammonium formate (pH 9.0) and B, CH_3_CN. The flow rate was 0.7 ml min^−1^ with a gradient of 10% to 50% B, for 60 min. Fractions containing the natural thiooctose substrate were monitored using an Acquity UPLC system with a 2996 PDA detection system (194–600 nm), connected to an LCT Premier XE time-of-flight mass spectrometer (Waters). The sample was loaded onto an Acquity Premier BEH amide LC column (2.1 × 50 mm, 1.7 µm), maintained at 40 °C. A two-component mobile phase—solvents A (20 mM ammonium formate (pH 4.75):CH_3_CN = 1:1 vol/vol%) and B (20 mM ammonium formate (pH 4.75):CH_3_CN = 1:9 vol/vol%)—was used for separation. Elution was performed at a flow rate of 0.4 ml min^−1^ with the following gradient (min, % of B: 1 min, 99%; 7 min, 1.0%; 9 min, 1.0%; 10 min, 99%), followed by 2 min of equilibration with 99% solvent B.

### Large-scale reaction for structural determination of 9

The large-scale reaction of the CcbF Y195G variant with substrate **4** was performed in a 5-ml reaction mixture containing 100 mM KPi (pH 8.0), 400 μM substrate **4**, 0.5 mM EDTA, 0.5 mM TCEP, 0.5 mM PLP and 20 μM enzyme, incubated overnight at 37 °C. After incubation, the reaction was quenched with an equal volume of methanol. For isolation of product **8**, the reaction was purified by HPLC with an LC-20AD and SPD-M20A system (Shimadzu Corporation) equipped with an XSelect HSS T3 OBD Prep column (10 × 250 mm, 5 μm; Waters). Gradient elution was performed with solvent A (NH_4_OH, pH 9.0) and solvent B (CH_3_CN) at a flow rate of 3 ml min^−1^ (*T* = 0 min, 20% B; *T* = 5 min, 20% B; *T* = 20 min, 100% B; *T* = 25 min, 100% B; *T* = 25.5 min, 20% B). Finally, 0.5 mg of compound **9** was obtained from 1 mg of **4**. ^1^H NMR (900 MHz, CD_3_OD): δ 5.45 (d, J = 5.5, 1H), 4.32 (d, J = 6.8, 1H), 4.14 (m, 2H), 4.05 (d, J = 3.0 Hz, 1H), 3.97 (dq, J = 6.4, 6.3 Hz 1H), 3.62 (dd, J = 3.0,10.1 Hz, 1H), 3.31 (d, J = 14.8 Hz, 1H), 3.21 (dd, J = 6.0, 8.8 Hz, 1H), 3.20 (d, J = 14.8 Hz, 1H), 2.95 (dd, J = 4.3, 10.6 Hz, 1H), 2.38 (s, 3H), 2.25 (m, 1H), 2.07 (dd, J = 8.8, 9.9 Hz, 1H), 2.00 (m, 1H), 1.81 (m, 1H), 1.32–1.38 (m, 4H), 1.22 (d, J = 6.3 Hz, 1H), 0.94 (t, J = 5.6 Hz, 1H), ^13^C NMR (225 MHz, CD_3_OD): 176.9, 173.6, 86.5, 70.4, 69.3, 69.3, 68.7, 67.9, 66.8, 62.4, 55.1, 40.4, 37.5, 37.4, 35.5, 32.2, 21.3, 18.6, 13.2. ESI-HRMS calculated for C_19_H_35_N_3_O_7_S [M + H]^+^ = 450.2268, found: 450.2286.

### Crystallization and structure determination

Crystals of LmbF and CcbF were obtained after one day at 20 °C. All crystals were obtained using the sitting-drop vapour-diffusion method with the following reservoir solutions: LmbF, 0.1 M MES, pH 6.5, 27% PEG 4000, 0.8 M LiCl; CcbF, 0.1 M Tris-Cl, pH 8.5, 31% PEG 4000, 0.16 M NaOAc. The crystals were transferred into cryoprotectant solution (reservoir solution with 25% (vol/vol) glycerol), then flash-cooled at −173 °C in a nitrogen-gas stream. The X-ray diffraction datasets were collected at BL-1A (Photon Factory), using a beam wavelength of 1.1 Å. Diffraction datasets were processed and scaled using the XDS program package^52^ and Aimless in CCP4 (ref. ^53^). The initial phases of the LmbF and CcbF structures were determined by molecular replacement, using the model structures of LmbF and CcbF, constructed by ColabFold^54^, as the search models, respectively. Molecular replacement was performed with Phaser^55^ in PHENIX^56^. The initial phases were further calculated with AutoBuild in PHENIX^56^. The structures were modified manually with Coot^57^ and refined with PHENIX.refine^58^. The final crystal data and intensity statistics are summarized in Extended Data Table 1. The Ramachandran statistics are as follows: 98.4% favoured, 1.6% allowed for LmbF; 97.9% favoured, 2.1% allowed for CcbF. A structural similarity search was performed, using the Dali program server^59^. All crystallographic figures were prepared with PyMOL (DeLano Scientific, http://www.pymol.org).

### Docking and MD simulations of LmbF and CcbF

Despite the availability of numerous docking simulation software tools, such as Autodock Vina^60^, the accurate prediction of protein–ligand complex coordinates is challenging, especially when only the *apo* form is available. This difficulty arises mainly because the software tools cannot consider the conformational changes of the main and side chains between the *apo* and *holo* forms. Meanwhile, in our study, the predicted coordinates of the PLP moiety of the ligand should not deviate substantially from those observed in our LmbF and CcbF crystal structures. To circumvent this, we performed only MD-based energy minimization and structural relaxation steps to generate the initial structure of the LmbF/CcbF–ligand complex after docking the ligand manually, instead of conventional docking simulations.

To obtain parameters for MD simulations, the structures of the external aldimine intermediate and the quinonoid intermediate of substrate **4** were optimized using Gaussian 16 Rev. C.02 (ref. ^61^) at the B3LYP level of theory^62–65^ with the 6-31+G(d,p) basis set. Their partial charges were obtained by restrained electrostatic potential (RESP), using the ANTECHAMBER^66^ module implemented in AmberTools22 (ref. ^67^). The general AMBER force field 2 (GAFF2)^68^ was used for the mechanical parameters of the ligands.

Ligand docking into the crystal structures was conducted manually according to the following procedure. First, the pyridine ring moiety of the external aldimine intermediate was superimposed on the position of the one observed in the respective crystal structures of LmbF and CcbF. The orientation of the Cα–H bond of the cysteine moiety in LmbF was arranged as shown in Fig. 3. Second, considering the voids present at the dimer interface, both dihedral angles −90° or −180° are possible for N–Cα–Cβ–S (Supplementary Fig. 11). However, we adopted −90° as −180° is unfavourable for the reaction, because the distance between the ε-amino group of Lys270 and the S atom was elongated. The rest of the ligand was modelled using the optimized substructure obtained by Gaussian 16. In CcbF, in addition to this conformation, the intermediate was docked so that the Cα–COO^–^ bond was perpendicular to the pyridine ring, as shown in Fig. 4.

After adding H atoms, the modelled LmbF and CcbF complexes were fully solvated in the OPC water model^69^ in a cubic periodic box, then neutralized by adding Na^+^ and Cl^–^ ions via the Amber LEaP module^70^. The ff19SB force field^71^ was used for the protein. Short-range van der Waals and electrostatic interactions were cut off beyond 10 Å, and the particle mesh Ewald (PME) method^72^ was used for long-range electrostatic interactions. The system was first relaxed using 200 steps of the steepest descent minimization, with a 1,000 kcal mol^−1^ Å^−2^ constraint applied to the heavy atoms of the protein. Subsequently, the entire system was subjected to 200 steps of the steepest descent minimization without restraints. Next, to gradually heat the system, 1-ns MD simulations were performed at 300 K and 1.0 × 10^5^ Pa under the NPT ensemble. During equilibrations, the SHAKE algorithm^73^ was used to constrain the bonds including H atoms, and the integration time step was set to 2 fs. The Berendsen weak coupling algorithm^74^ was used to maintain constant temperature and pressure. We repeated the minimization and equilibration steps twice to resolve steric clashes between the ligand and the protein in the initial modelled structure, especially around LmbF-Leu83 and -Trp150. After equilibration, 50-ns conventional production runs were performed. Equilibrations and production runs were carried out using the PMEMD module^75^ of AMBER 22 (ref. ^67^). The distance and dihedral calculations were carried out using CPPTRAJ^76^.

### Reporting summary

Further information on research design is available in the Nature Portfolio Reporting Summary linked to this Article.

## Online content

Any methods, additional references, Nature Portfolio reporting summaries, source data, extended data, supplementary information, acknowledgements, peer review information; details of author contributions and competing interests; and statements of data and code availability are available at 10.1038/s41557-024-01687-7.

## Supplementary information

Supplementary Information Supplementary Table 1 and Figs. 1–13.

Reporting Summary

Supplementary Data 1 Primer list used in this study.

## Source data

Source Data Fig. 3: Raw data for calculated distances and angles. Source Data Fig. 4: Raw data for calculated distances and angles. Source Data Fig. 5: Raw data for LC-MS chart. Source Data Extended Data Fig./Table 2: Raw data for calculated distances and angles. Source Data Extended Data Fig./Table 3: Raw data for calculated distances and angles. Source Data Extended Data Fig./Table 4: Raw data for LC-MS chart. Source Data Extended Data Fig./Table 5: Raw data for LC-MS chart. Source Data Extended Data Fig./Table 7: Raw data for calculated distances and angles. Source Data Extended Data Fig./Table 8: Raw data for LC-MS chart. Source Data Extended Data Fig./Table 9: Raw data for LC-MS chart.

## Figures and Tables

**Extended Data Table 1:**
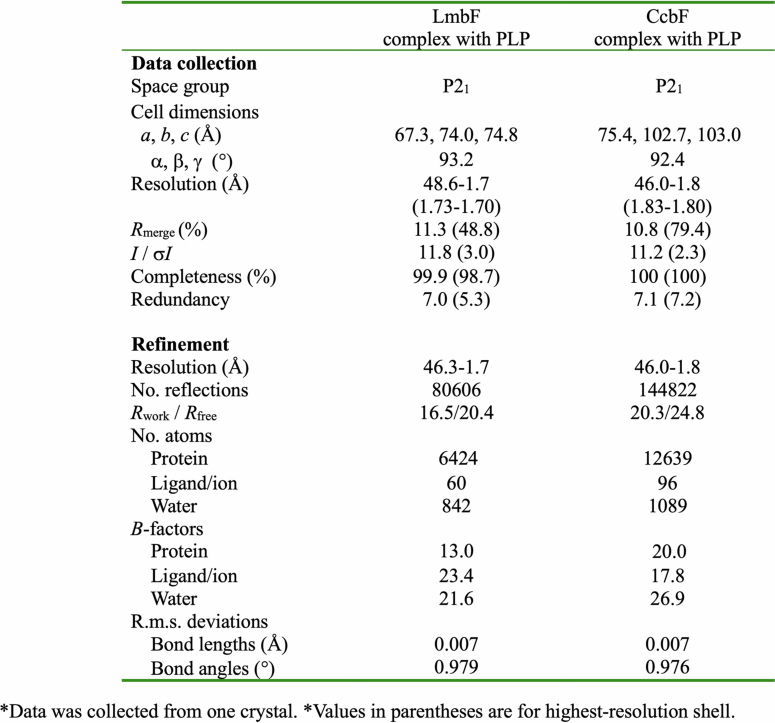
Data collection and refinement statistics

## Data Availability

Data supporting the findings of this study are available within the Article and its Supplementary Information and source files. The MSA files (in a3m format) and predicted LmbF and CcbF models using the MSAs (in pdb format) are freely available at Zenodo (10.5281/zenodo.13309462)^77^. The crystallographic data that support the findings of this study are available from the Protein Data Bank (http://www.rcsb.org). The coordinates and structure factor amplitudes for LmbF and CcbF have been deposited under accession codes 8KDL and 8KDK, respectively. Source data are provided with this paper.

## References

[1] Mason, D. J. & Lewis, C. Biological activity of the lincomycin-related antibiotics. *Antimicrob. Agents Chemother.* **10**, 7–12 (1964). 14288036

[2] Owen, S. P., Bhuyan, B. K. & Kupiecki, F. P. Discovery and biological properties of *cis*-β-carboxyacrylamidine, a new cytotoxic agent. *Antimicrob. Agents Chemother.* **5**, 808–811 (1965). 5883502

[3] Hoeksema, H. & Celesticetin, V. The structure of celesticetin. *J. Am. Chem. Soc.* **90**, 755–757 (1968). 5638307 10.1021/ja01005a036

[4] Hanada, M., Tsunakawa, M., Tomita, K., Tsukiura, H. & Kawaguchi, H. Antibiotic Bu-2545, a new member of the celesticetin-lincomycin class. *J. Antibiot.* **33**, 751–753 (1980). 10.7164/antibiotics.33.751 7410215

[5] Lin, C. I., McCarty, R. M. & Liu, H. W. The biosynthesis of nitrogen-, sulfur- and high-carbon chain-containing sugars. *Chem. Soc. Rev.* **42**, 4377–4407 (2013). 23348524 10.1039/c2cs35438a PMC3641179

[6] Janata, J. et al. Lincosamide synthetase—a unique condensation system combining elements of nonribosomal peptide synthetase and mycothiol metabolism. *PLoS ONE* **10**, e0118850 (2015). 25741696 10.1371/journal.pone.0118850 PMC4351081

[7] Spizek, J. & Rezanka, T. Lincosamides: chemical structure, biosynthesis, mechanism of action, resistance and applications. *Biochem. Pharmacol.* **133**, 20–28 (2017). 27940264 10.1016/j.bcp.2016.12.001

[8] Zhang, D., Tang, Z. & Liu, W. Biosynthesis of lincosamide antibiotics: reactions associated with degradation and detoxification pathways play a constructive role. *Acc. Chem. Res.* **51**, 1496–1506 (2018). 29792672 10.1021/acs.accounts.8b00135

[9] Schlünzen, F. et al. Structural basis for the interaction of antibiotics with the peptidyl transferase centre in eubacteria. *Nature* **413**, 814–821 (2001). 11677599 10.1038/35101544

[10] Tenson, T. & Ehrenberg, M. Regulatory nascent peptides in the ribosomal tunnel. *Cell* **108**, 591–594 (2002). 11893330 10.1016/s0092-8674(02)00669-4

[11] Hoeksema, H. et al. Chemical studies on lincomycin. I. The structure of lincomycin. *J. Am. Chem. Soc.* **86**, 4223–4224 (1964).

[12] Janata, J., Kamenik, Z., Gazak, R., Kadlcik, S. & Najmanova, L. Biosynthesis and incorporation of an alkylproline-derivative (APD) precursor into complex natural products. *Nat. Prod. Rep.* **35**, 257–289 (2018). 29517100 10.1039/c7np00047b

[13] Kadlcik, S., Kamenik, Z., Vasek, D., Nedved, M. & Janata, J. Elucidation of salicylate attachment in celesticetin biosynthesis opens the door to create a library of more efficient hybrid lincosamide antibiotics. *Chem. Sci.* **8**, 3349–3355 (2017). 28507704 10.1039/c6sc04235j PMC5416915

[14] Zhao, Q., Wang, M., Xu, D., Zhang, Q. & Liu, W. Metabolic coupling of two small-molecule thiols programs the biosynthesis of lincomycin A. *Nature* **518**, 115–119 (2015). 25607359 10.1038/nature14137

[15] Wang, M., Zhao, Q., Zhang, Q. & Liu, W. Differences in PLP-dependent cysteinyl processing lead to diverse *S*-functionalization of lincosamide antibiotics. *J. Am. Chem. Soc.* **138**, 6348–6351 (2016). 27171737 10.1021/jacs.6b01751

[16] Ushimaru, R., Lin, C.-I., Sasaki, E. & Liu, H.-W. Characterization of enzymes catalyzing transformations of cysteine *S*-conjugated intermediates in the lincosamide biosynthetic pathway. *ChemBioChem* **17**, 1606–1611 (2016). 27431934 10.1002/cbic.201600223 PMC5253346

[17] Kamenik, Z. et al. Deacetylation of mycothiol-derived ‘waste product’ triggers the last biosynthetic steps of lincosamide antibiotics. *Chem. Sci.* **7**, 430–435 (2016). 28791100 10.1039/c5sc03327f PMC5518657

[18] Wang, S. A. et al. Studies of lincosamide formation complete the biosynthetic pathway for lincomycin A. *Proc. Natl Acad. Sci. USA* **117**, 24794–24801 (2020). 32958639 10.1073/pnas.2009306117 PMC7547251

[19] Hoffarth, E. R., Rothchild, K. W. & Ryan, K. S. Emergence of oxygen- and pyridoxal phosphate-dependent reactions. *FEBS J.* **287**, 1403–1428 (2020). 32142210 10.1111/febs.15277

[20] Eliot, A. C. & Kirsch, J. F. Pyridoxal phosphate enzymes: mechanistic, structural and evolutionary considerations. *Annu. Rev. Biochem.* **73**, 383–415 (2004). 15189147 10.1146/annurev.biochem.73.011303.074021

[21] Dunathan, H. C. Conformation and reaction specificity in pyridoxal phosphate enzymes. *Proc. Natl Acad. Sci. USA* **55**, 712–716 (1966). 5219675 10.1073/pnas.55.4.712 PMC224217

[22] Toney, M. D. Reaction specificity in pyridoxal phosphate enzymes. *Arch. Biochem. Biophys.* **433**, 279–287 (2005). 15581583 10.1016/j.abb.2004.09.037

[23] Du, Y. L. & Ryan, K. S. Pyridoxal phosphate-dependent reactions in the biosynthesis of natural products. *Nat. Prod. Rep.* **36**, 430–457 (2019). 30183796 10.1039/c8np00049b

[24] Torrens-Spence, M. P. et al. Biochemical evaluation of the decarboxylation and decarboxylation-deamination activities of plant aromatic amino acid decarboxylases. *J. Biol. Chem.* **288**, 2376–2387 (2013). 23204519 10.1074/jbc.M112.401752 PMC3554908

[25] Torrens-Spence, M. P. et al. Structural basis for divergent and convergent evolution of catalytic machineries in plant aromatic amino acid decarboxylase proteins. *Proc. Natl Acad. Sci. USA* **117**, 10806–10817 (2020). 32371491 10.1073/pnas.1920097117 PMC7245119

[26] Liang, J., Han, Q., Ding, H. & Li, J. Biochemical identification of residues that discriminate between 3,4-dihydroxyphenylalanine decarboxylase and 3,4-dihydroxyphenylacetaldehyde synthase-mediated reactions. *Insect Biochem. Mol. Biol.* **91**, 34–43 (2017). 29037755 10.1016/j.ibmb.2017.10.001

[27] Vavricka, C. J. et al. Mechanism-based tuning of insect 3,4-dihydroxyphenylacetaldehyde synthase for synthetic bioproduction of benzylisoquinoline alkaloids. *Nat. Commun.* **10**, 2015 (2019). 31043610 10.1038/s41467-019-09610-2 PMC6494836

[28] Chun, S. W. & Narayan, A. R. H. Biocatalytic, stereoselective deuteration of α-amino acids and methyl esters. *ACS Catal.* **10**, 7413–7418 (2020). 34430066 10.1021/acscatal.0c01885 PMC8382264

[29] John, R. A. Pyridoxal phosphate-dependent enzymes. *Biochim. Biophys. Acta* **1248**, 81–96 (1995). 7748903 10.1016/0167-4838(95)00025-p

[30] Walsh, C. T. & Tang, Y. *The Chemical Biology of Human Vitamins* (Royal Society of Chemistry, 2018).

[31] Barra, L. et al. β-NAD as a building block in natural product biosynthesis. *Nature* **600**, 754–758 (2021). 34880494 10.1038/s41586-021-04214-7

[32] Gao, J. et al. A pyridoxal 5′-phosphate-dependent Mannich cyclase. *Nat. Catal.* **6**, 476–486 (2023). 10.1002/cbic.202300561 PMC10874886 37779345

[33] Ringel, M. T., Dräger, G. & Brüser, T. PvdN enzyme catalyzes a periplasmic pyoverdine modification. *J. Biol. Chem.* **291**, 23929–23938 (2016). 27703013 10.1074/jbc.M116.755611 PMC5104919

[34] Huang, Y. et al. Pyridoxal-5′-phosphate as an oxygenase cofactor: discovery of a carboxamide-forming, α-amino acid monooxygenase-decarboxylase. *Proc. Natl Acad. Sci. USA* **115**, 974–979 (2018). 29343643 10.1073/pnas.1718667115 PMC5798378

[35] Liu, S. et al. Molecular and structural basis for Cγ–C bond formation by PLP-dependent enzyme Fub7. *Angew. Chem. Int. Ed.* **63**, e202317161 (2024). 10.1002/anie.202317161 PMC10947850 38308582

[36] Steffen-Munsberg, F. et al. Bioinformatic analysis of a PLP-dependent enzyme superfamily suitable for biocatalytic applications. *Biotechnol. Adv.* **33**, 566–604 (2015). 25575689 10.1016/j.biotechadv.2014.12.012

[37] Rocha, J. F., Pina, A. F., Sousa, S. F. & Cerqueira, N. M. F. S. A. PLP-dependent enzymes as important biocatalysts for the pharmaceutical, chemical and food industries: a structural and mechanistic perspective. *Catal. Sci. Technol.* **9**, 4864–4876 (2019).

[38] Chen, M., Liu, C.-T. & Tang, Y. Discovery and biocatalytic application of a PLP-dependent amino acid γ-substitution enzyme that catalyzes C–C bond formation. *J. Am. Chem. Soc.* **142**, 10506–10515 (2020). 10.1021/jacs.0c03535 PMC7335459 32434326

[39] Clausen, T., Huber, R., Messerschmidt, A., Pohlenz, H.-D. & Laber, B. Slow-binding inhibition of *Escherichia coli* cystathionine β-lyase by l-aminoethoxyvinylglycine: a kinetic and X-ray study. *Biochemistry* **36**, 12633–12643 (1997). 9376370 10.1021/bi970630m

[40] Matoba, Y. et al. Catalytic specificity of the *Lactobacillus plantarum* cystathionine γ-lyase presumed by the crystallographic analysis. *Sci. Rep.* **10**, 14886 (2020). 32913258 10.1038/s41598-020-71756-7 PMC7483736

[41] Irani, S. et al. Snapshots of C-S cleavage in Egt2 reveals substrate specificity and reaction mechanism. *Cell Chem. Biol.* **25**, 519–529 (2018). 29503207 10.1016/j.chembiol.2018.02.002 PMC5959753

[42] Song, H. et al. Mechanistic studies of a novel C-S lyase in ergothioneine biosynthesis: the involvement of a sulfenic acid intermediate. *Sci. Rep.* **5**, 11870 (2015). 26149121 10.1038/srep11870 PMC4493562

[43] Meng, S. et al. Thiocysteine lyases as polyketide synthase domains installing hydropersulfide into natural products and a hydropersulfide methyltransferase. *Nat. Commun.* **12**, 5672 (2021). 34584078 10.1038/s41467-021-25798-8 PMC8479088

[44] Burkhard, P., Dominici, P., Borri-Voltattorni, C., Jansonius, J. N. & Malashkevich, V. N. Structural insight into Parkinson’s disease treatment from drug-inhibited DOPA decarboxylase. *Nat. Struct. Biol.* **8**, 963–967 (2001). 11685243 10.1038/nsb1101-963

[45] Chellam Gayathri, S. & Manoj, N. Structural insights into the mechanism of internal aldimine formation and catalytic loop dynamics in an archaeal Group II decarboxylase. *J. Struct. Biol.* **208**, 137–151 (2019). 31445086 10.1016/j.jsb.2019.08.009

[46] Han, L. et al. *Streptomyces wadayamensis* MppP is a PLP-dependent oxidase, not an oxygenase. *Biochemistry* **57**, 3252–3264 (2018). 29473729 10.1021/acs.biochem.8b00130

[47] Du, Y. L. et al. A pyridoxal phosphate-dependent enzyme that oxidizes an unactivated carbon–carbon bond. *Nat. Chem. Biol.* **12**, 194–199 (2016). 26807714 10.1038/nchembio.2009

[48] Hoffarth, E. R. et al. A shared mechanistic pathway for pyridoxal phosphate-dependent arginine oxidases. *Proc. Natl Acad. Sci. USA* **118**, e2012591118 (2021). 34580201 10.1073/pnas.2012591118 PMC8501904

[49] Cai, W. et al. The biosynthesis of capuramycin-type antibiotics: identification of the A-102395 biosynthetic gene cluster, mechanism of self-resistance and formation of uridine-5′-carboxamide. *J. Biol. Chem.* **290**, 13710–13724 (2015). 25855790 10.1074/jbc.M115.646414 PMC4447950

[50] Drake, E. J. & Gulick, A. M. 1.2 Å resolution crystal structure of the periplasmic aminotransferase PvdN from *Pseudomonas aeruginosa*. *Acta Crystallogr. F* **72**, 403–408 (2016). 10.1107/S2053230X16006257 PMC4854569 27139833

[51] Daniel-Ivad, P. G., Van Lanen, S. & Ryan, K. S. Structure of the oxygen, pyridoxal phosphate-dependent capuramycin biosynthetic protein Cap15. *Biochemistry* **62**, 2611–2621 (2023). 37556254 10.1021/acs.biochem.3c00216

[52] Kabsch, W. XDS. *Acta Crystallogr. D* **66**, 125–132 (2010). 10.1107/S0907444909047337 PMC2815665 20124692

[53] Evans, P. R. & Murshudov, G. N. How good are my data and what is the resolution? *Acta Crystallogr. D* **69**, 1204–1214 (2013). 10.1107/S0907444913000061 PMC3689523 23793146

[54] Mirdita, M. et al. ColabFold: making protein folding accessible to all. *Nat. Methods* **19**, 679–682 (2022). 35637307 10.1038/s41592-022-01488-1 PMC9184281

[55] McCoy, A. J. et al. Phaser crystallographic software. *J. Appl. Crystallogr.* **40**, 658–674 (2007). 19461840 10.1107/S0021889807021206 PMC2483472

[56] Adams, P. D. et al. PHENIX: a comprehensive Python-based system for macromolecular structure solution. *Acta Crystallogr. D* **66**, 213–221 (2010). 10.1107/S0907444909052925 PMC2815670 20124702

[57] Emsley, P. & Cowtan, K. Coot: model-building tools for molecular graphics. *Acta Crystallogr. D* **60**, 2126–2132 (2004). 10.1107/S0907444904019158 15572765

[58] Afonine, P. V. et al. Towards automated crystallographic structure refinement with phenix.refine. *Acta Crystallogr. D* **68**, 352–367 (2012). 10.1107/S0907444912001308 PMC3322595 22505256

[59] Holm, L. & Rosenström, P. Dali server: conservation mapping in 3D. *Nucleic Acids Res.* **38**, W545–W549 (2010). 20457744 10.1093/nar/gkq366 PMC2896194

[60] Eberhardt, J., Santos-Martins, D., Tillack, A. F. & Forli, S. AutoDock Vina 1.2.0: new docking methods, expanded force field and Python bindings. *J. Chem. Inf. Model.* **61**, 3891–3898 (2021). 34278794 10.1021/acs.jcim.1c00203 PMC10683950

[61] Frisch, M. J. et al. *Gaussian 16 Revision C.01* (Gaussian, 2016).

[62] Becke, A. D. A new mixing of Hartree-Fock and local density‐functional theories. *J. Chem. Phys.* **98**, 1372–1377 (1993).

[63] Becke, A. D. Density‐functional thermochemistry. III. The role of exact exchange. *J. Chem. Phys.* **98**, 5648–5652 (1993).

[64] Becke, A. D. Density-functional exchange-energy approximation with correct asymptotic behavior. *Phys. Rev. A* **38**, 3098–3100 (1988). 10.1103/physreva.38.3098 9900728

[65] Stephens, P. J., Devlin, F. J., Chabalowski, C. F. & Frisch, M. J. Ab initio calculation of vibrational absorption and circular dichroism spectra using density functional force fields. *J. Phys. Chem.* **98**, 11623–11627 (1994).

[66] Wang, J., Wang, W., Kollman, P. A. & Case, D. A. Automatic atom type and bond type perception in molecular mechanical calculations. *J. Mol. Graph. Model.* **25**, 247–260 (2006). 10.1016/j.jmgm.2005.12.005 16458552

[67] Case, D. A. et al. *AMBER 22* (Univ. California, 2022).

[68] He, X., Man, V. H., Yang, W., Lee, T.-S. & Wang, J. A fast and high-quality charge model for the next generation general AMBER force field. *J. Chem. Phys.* **153**, 114502 (2020). 32962378 10.1063/5.0019056 PMC7728379

[69] Izadi, S., Anandakrishnan, R. & Onufriev, A. V. Building water models: a different approach. *J. Phys. Chem. Lett.* **5**, 3863–3871 (2014). 25400877 10.1021/jz501780a PMC4226301

[70] Case, D. A. et al. The Amber biomolecular simulation programs. *J. Comput. Chem.* **26**, 1668–1688 (2005). 16200636 10.1002/jcc.20290 PMC1989667

[71] Tian, C. et al. ff19SB: amino-acid-specific protein backbone parameters trained against quantum mechanics energy surfaces in solution. *J. Chem. Theory Comput.* **16**, 528–552 (2020). 31714766 10.1021/acs.jctc.9b00591 PMC13071887

[72] Essmann, U. et al. A smooth particle mesh Ewald method. *J. Chem. Phys.* **103**, 8577–8593 (1995).

[73] Ryckaert, J.-P., Ciccotti, G. & Berendsen, H. J. C. Numerical integration of the cartesian equations of motion of a system with constraints: molecular dynamics of *n*-alkanes. *J. Comput. Phys.* **23**, 327–341 (1977).

[74] Berendsen, H. J. C., Postma, J. P. M., van Gunsteren, W. F., DiNola, A. & Haak, J. R. Molecular dynamics with coupling to an external bath. *J. Chem. Phys.* **81**, 3684–3690 (1984).

[75] Salomon-Ferrer, R., Götz, A. W., Poole, D., Le Grand, S. & Walker, R. C. Routine microsecond molecular dynamics simulations with AMBER on GPUs. 2. Explicit solvent particle mesh Ewald. *J. Chem. Theory Comput.* **9**, 3878–3888 (2013). 26592383 10.1021/ct400314y

[76] Roe, D. R. & Cheatham, T. E. III PTRAJ and CPPTRAJ: software for processing and analysis of molecular dynamics trajectory data. *J. Chem. Theory Comput.* **9**, 3084–3095 (2013). 26583988 10.1021/ct400341p

[77] Mori, T. et al. The MSA files in a3m format and predicted LmbF and CcbF models using the MSAs in pdb format. *Zenodo*10.5281/zenodo.13309462 (2024).

