# Supplementary material for: Molecular basis for the diversification of lincosamide biosynthesis by pyridoxal phosphate-dependent enzymes
Source: Nat Chem. 2024 Dec 6;17(2):256–64. doi: 10.1038/s41557-024-01687-7 (PMC11794154; doi:10.1038/s41557-024-01687-7)
Supplement: Supplementary file 1 — Supplementary Table 1 and Figs. 1–13. [file 41557_2024_1687_MOESM1_ESM.pdf]

nature chemistry

Supplementary information https://doi.org/10.1038/s41557-024-01687-7

Molecular basis for the diversification of lincosamide biosynthesis by pyridoxal phosphate-dependent enzymes

In the format provided by the authors and unedited

Table of contents

Supplementary Table

Supplementary Table 1. NMR table of **9**.

Supplementary Figures

Supplementary Figure 1. Proposed reaction mechanisms of LmbF and CcbF

Supplementary Figure 2. Superimposed view of LmbF and CcbF.

Supplementary Figure 3. Reactions of aromatic L-amino acid decarboxylases and amino acid aldehyde synthases.

Supplementary Figure 4. NMR analysis of **9**.

Supplementary Figure 5. Comparison of the active site of cystathionine β-lyases, Egt2, and LmbF.

Supplementary Figure 6. Comparison of the active site of decarboxylases and CcbF.

Supplementary Figure 7. Proposed reaction mechanism of S-acetamide formation by LmbF and CcbF variants.

Supplementary Figure 8. NMR data of **9**.

Supplementary Figure 9. HR-MS of **9**.

Supplementary Figure 10. Comparison of ^{1}H NMR spectra (amide moiety) among different solvents.

Supplementary Figure 11. Initial model of LmbF in complex with manually docked external aldimine models.

Supplementary Figure 12. SDS-PAGE of purified enzymes.

Supplementary Figure 13. Supplementary Figure 12. Stability analysis of LmbF, CcbF and their mutants.

Supplementary Tables

Supplementary Table 1. NMR table of **9**.

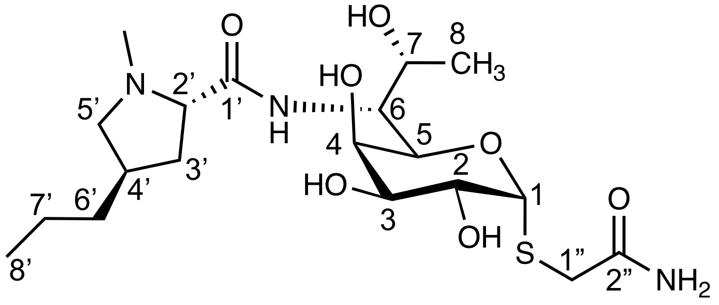

position	δH	m.	J_{HH} [Hz]	δC	COSY	HMBC
1	5.45	d	5.5	86.5	H-2	2, 5, 1"
2	4.14	m	-	67.9	H-1, 3	3
3	3.62	dd	3.0, 10.1	70.4	H-2, 4	1, 2
4	4.05	d	3.0	69.3	H-3	2, 3
5	4.32	d	6.8	69.3	H-6	1, 4, 6
6	4.14	m	-	55.1	H-5, 7	5, 7, 8, 1'
7	3.97	dq	6.4, 6.3	66.8	H-6, 8	6, 8
8	1.22	d	6.3	18.6	H-7	6, 7
N-CH3	2.38	s	-	40.4		2', 5'
1'				176.9		
2'	2.95	dd	4.3, 10.6	68.7	H-3'a, 3'b	*N*-CH3, 1', 3'
3'a	1.81	m	-	37.4	H-2', 4'	1', 4', 5', 6'
3'b	2.00	m	-		H-2', 4'	
4'	2.25	m	-	37.5	H-3'a, 3'b, 5'a, 5'b	3'
5'a	2.07	dd	8.8, 9.9	62.4	H-4	*N*-CH3, 2', 3'
5'b	3.21	dd	6.0, 8.8		H-4	
6'	1.32-1.38	m	-	35.5	H-7', 8'	4', 7'
7'	1.32-1.38	m	-	21.3	H-6', 8'	6'
8'	0.94	t	5.6	13.2	H-6', 7'	6', 7'
1"a	3.20	d	14.8	32.2		1, 2"
1"b	3.31	d	14.8			
2"				173.6		

Supplementary Figures

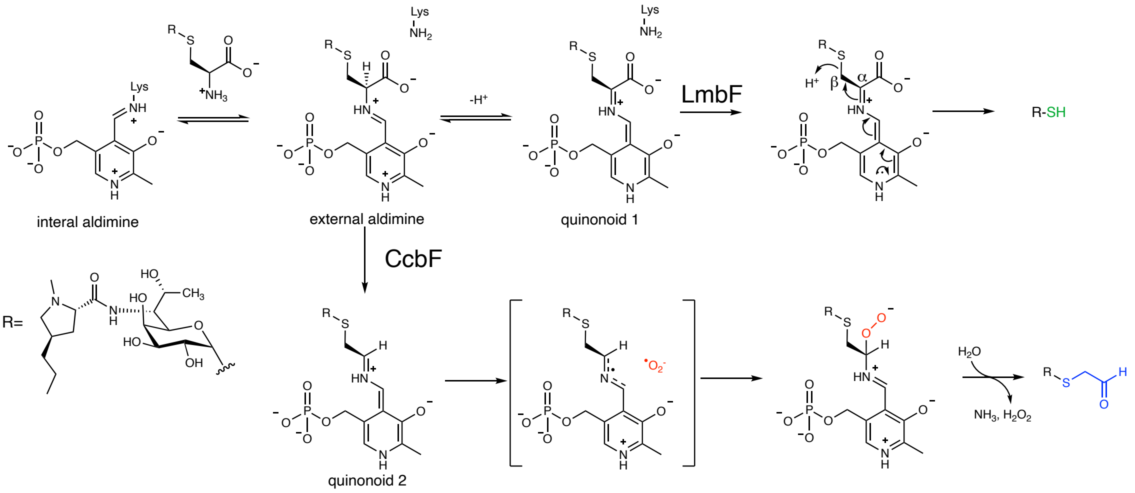

Supplementary Figure 1. Schematic mechanisms of LmbF and CcbF. LmbF catalyzes β-elimination through the deprotonation of a hydrogen atom from the Cα atom, while CcbF catalyzes decarboxylation from the external aldimine intermediate.

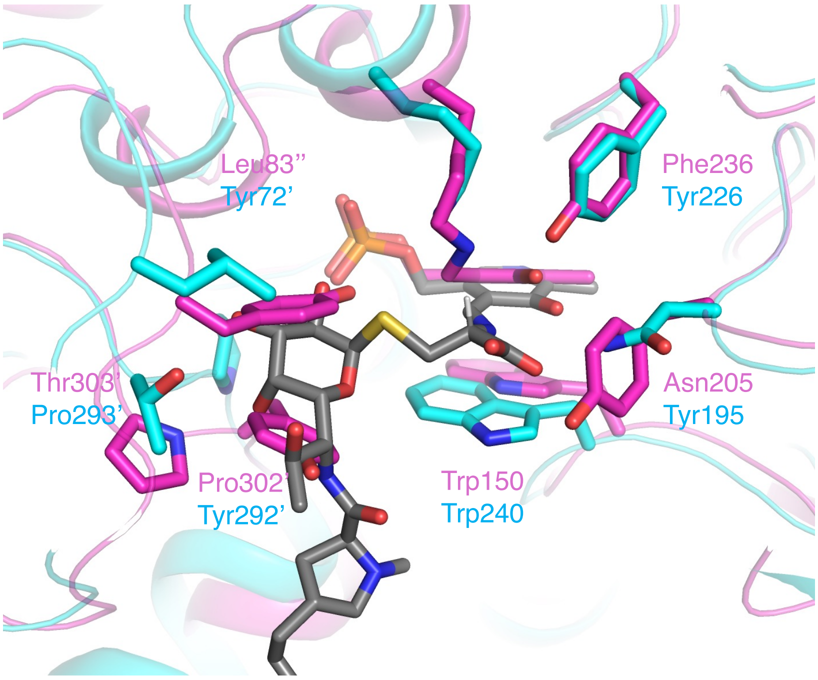

Supplementary Figure 2. **Superimposed view of LmbF and CcbF.** Comparison between the active site residues between LmbF (cyan) and CcbF (magenta). The docking model of external aldimine intermediate of **4** in LmbF is shown in gray.

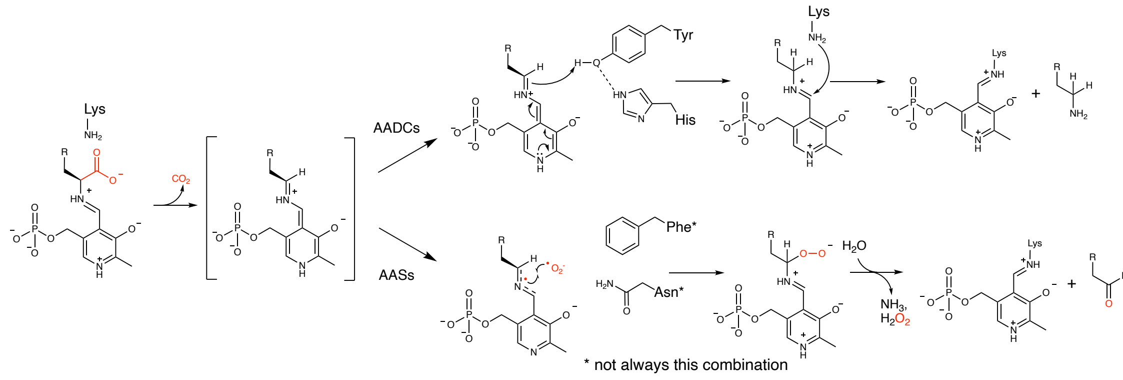

Supplementary Figure 3. Reactions of aromatic L-amino acid decarboxylases and amino acid aldehyde synthases.

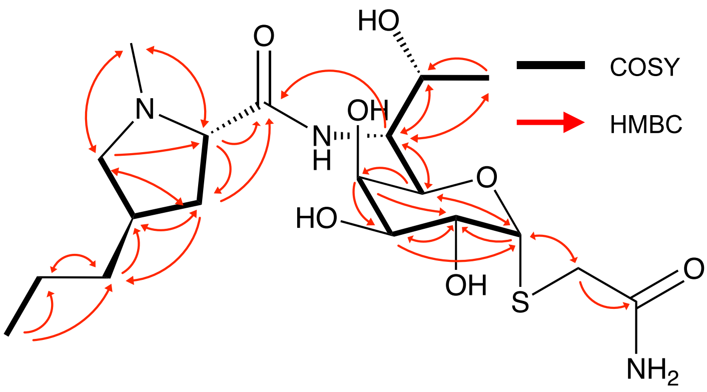

Supplementary Figure 4. **NMR analysis of 8.** COSY (bold) and key HMBC (arrows) correlations of **9**.

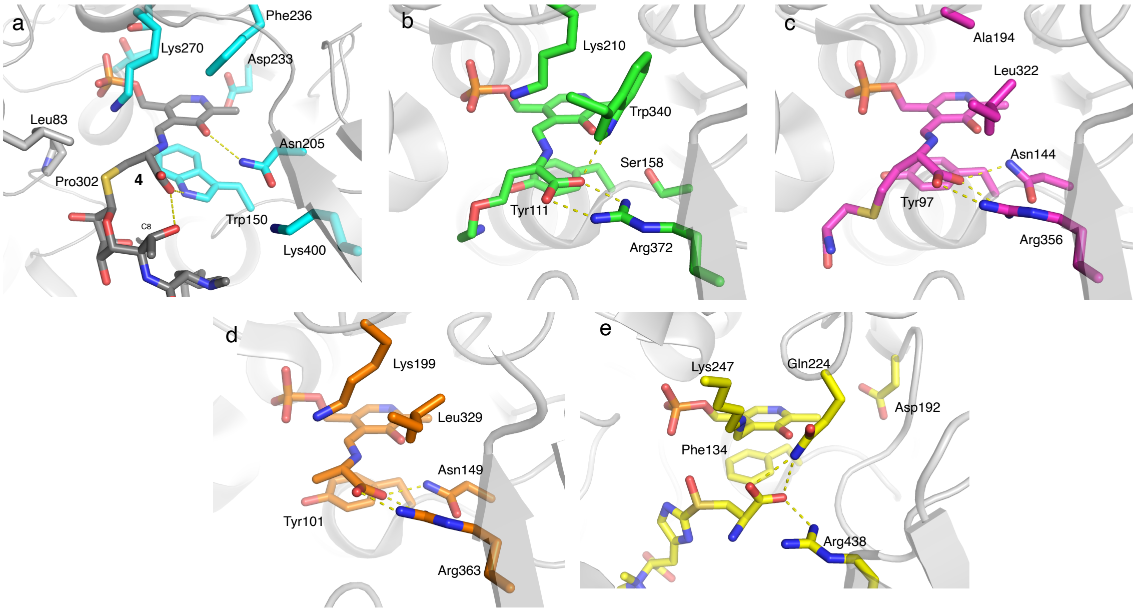

Supplementary Figure 5. **Comparison of the active sites of cystathionine β-lyases, Egt2, and LmbF**. Binding modes of ligands in the active sites of (a) the docking model of LmbF, (b) cystathionine β-lyase (CBL) from *Escherichia coli* (PDBID: 1CL2), (c) cystathionine γ-lyase from *Lactobacillus plantarum* (PDBID: 6LE4), (d) cystathionine β-lyase from *Legionella pneumophila* (PDBID: 6CJA), and (e) Egt2 (PDBID: 5V1X).

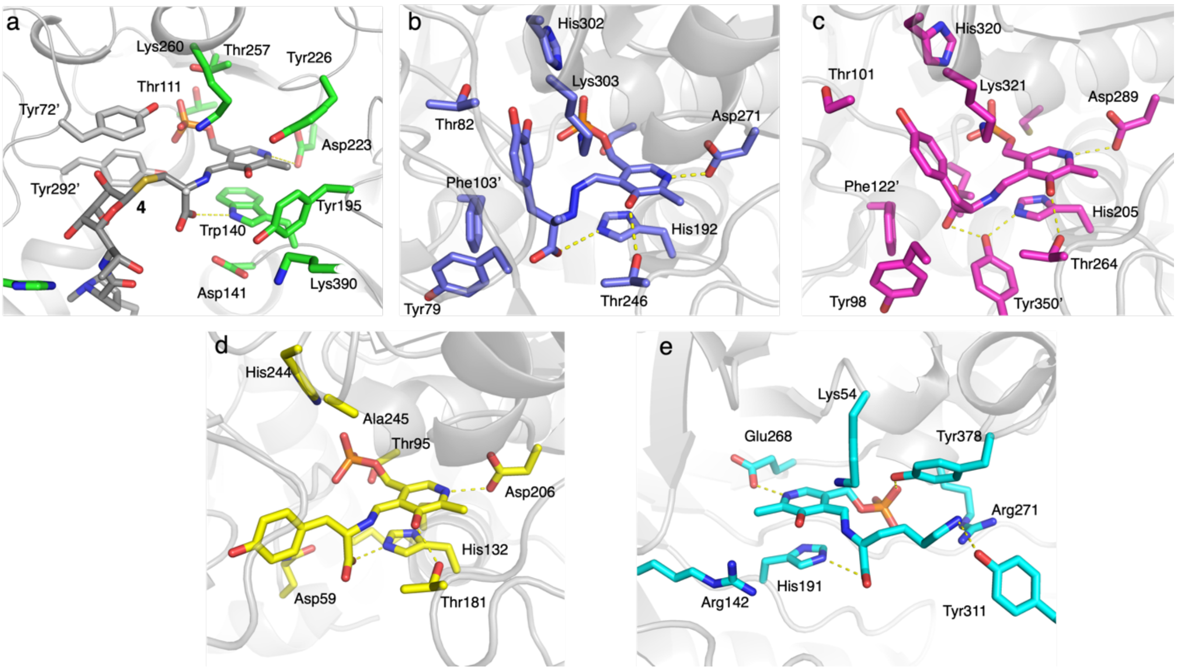

Supplementary Figure 6. **Comparison of the active sites of decarboxylases and CcbF.** Binding modes of ligands in the active sites of (a) the docking model of CcbF, (b) dopa decarboxylase from *Sus scrofa* (PDBID: 1JS3), (c) tyrosine decarboxylase from *Papaver somniferum* (PDBID: 6EEM), (d) K245A mutant of L-tyrosine decarboxylase from *Methanocaldococcus jannaschii* (PDBID: 6LDS), and (e) diaminopimelate decarboxylase from *Escherichia coli* (PDBID: 1KO0).

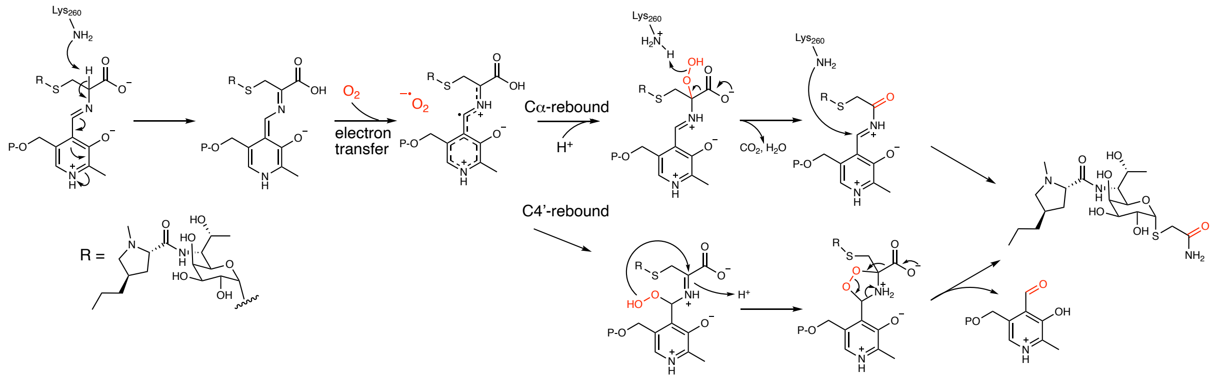

Supplementary Figure 7. **Proposed reaction mechanism of *S*-acetamide formation by LmbF and CcbF variants.**

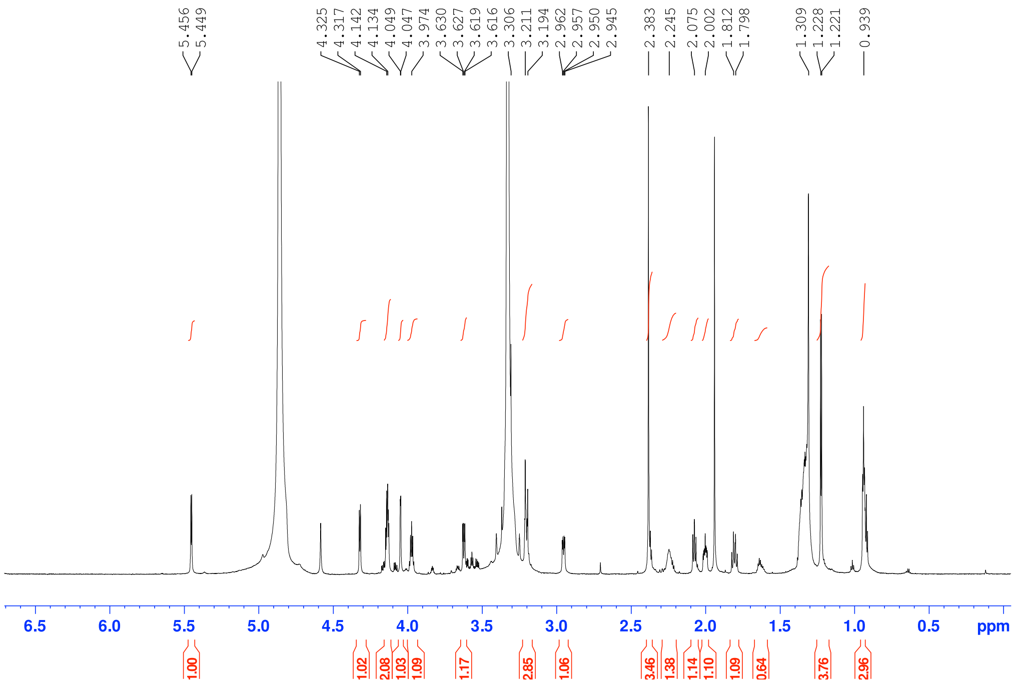

Supplementary Figure 8a. **^{1}H NMR spectrum of 9 in CD_3OD.**

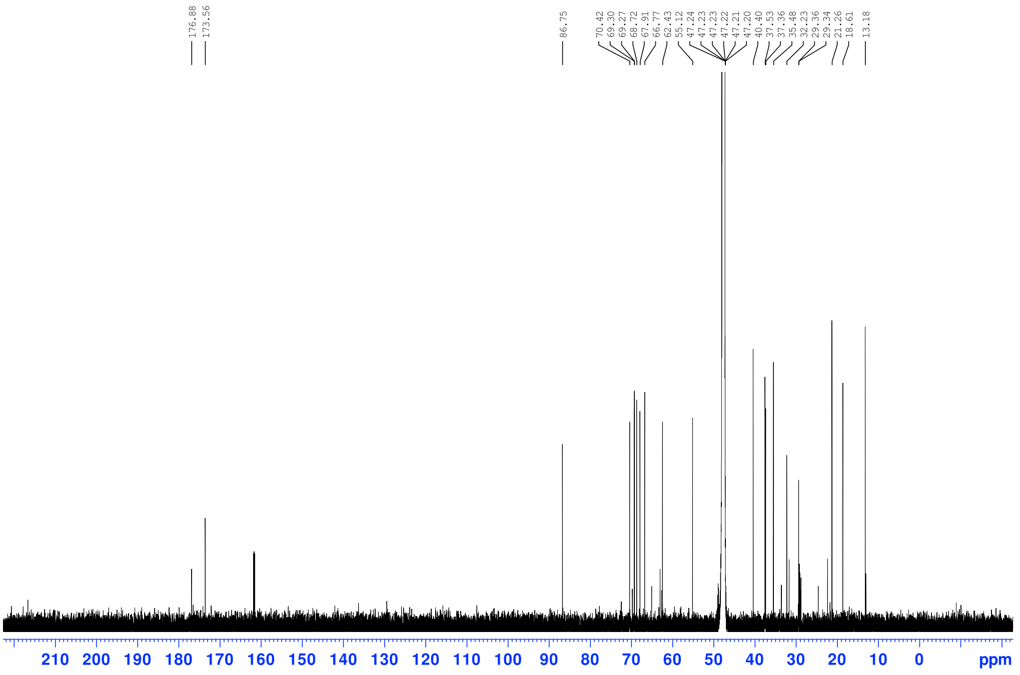

Supplementary Figure 8b. **^{13}C NMR spectrum of 9 in CD_3OD.**

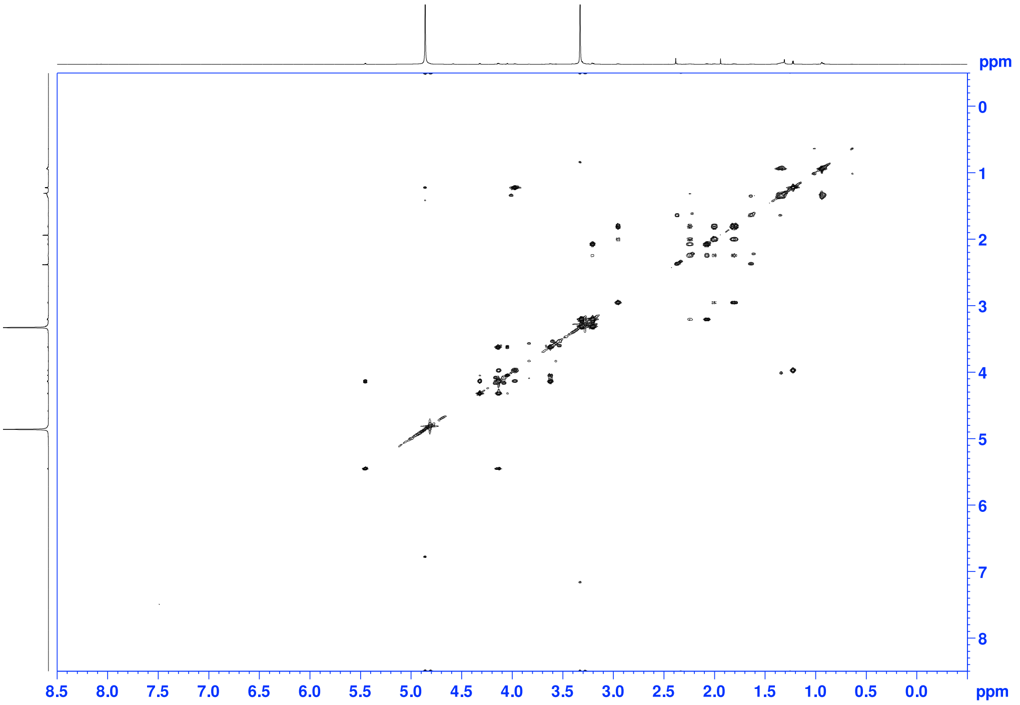

Supplementary Figure 8c. COSY spectrum of 9 in CD_3OD.

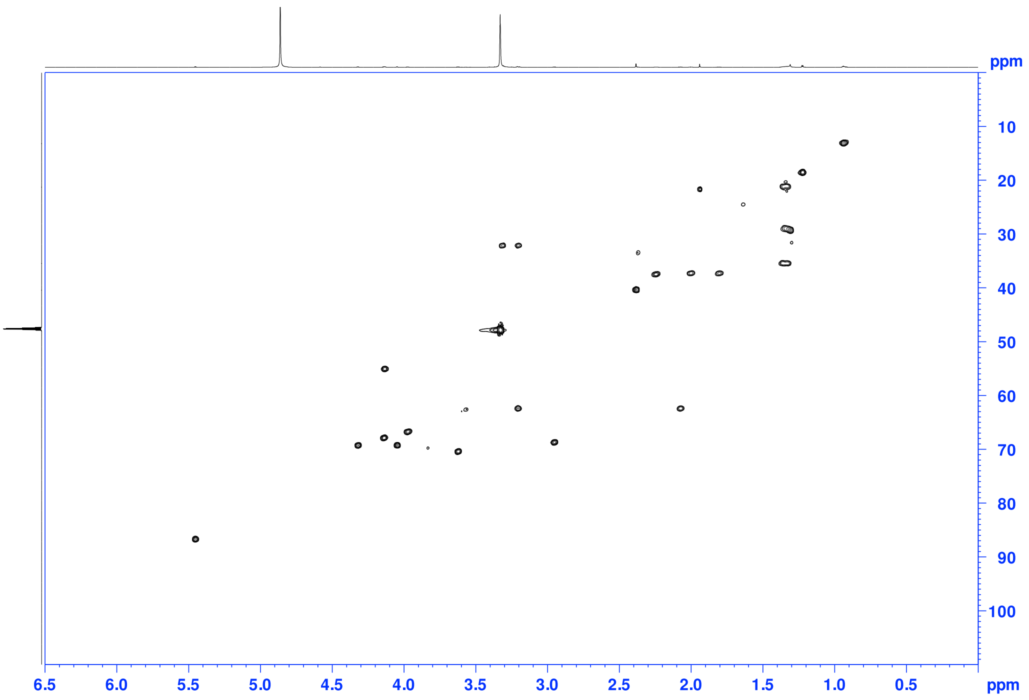

Supplementary Figure 8d. HSQC spectrum of 9 in CD_3OD.

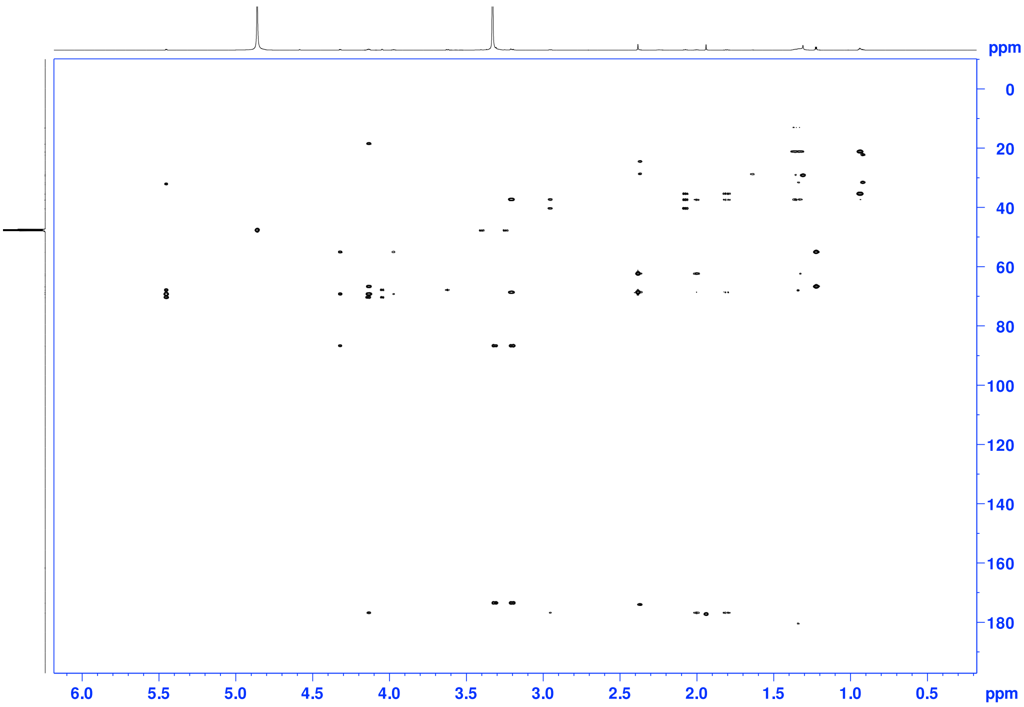

Supplementary Figure 8e. HMBC spectrum of 9 in CD_3OD.

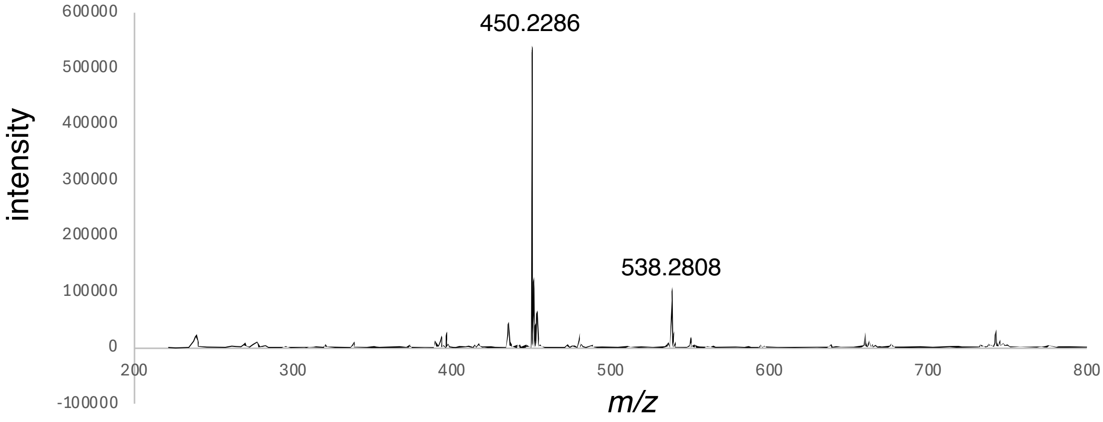

Supplementary Figure 9. HR-MS of 9.

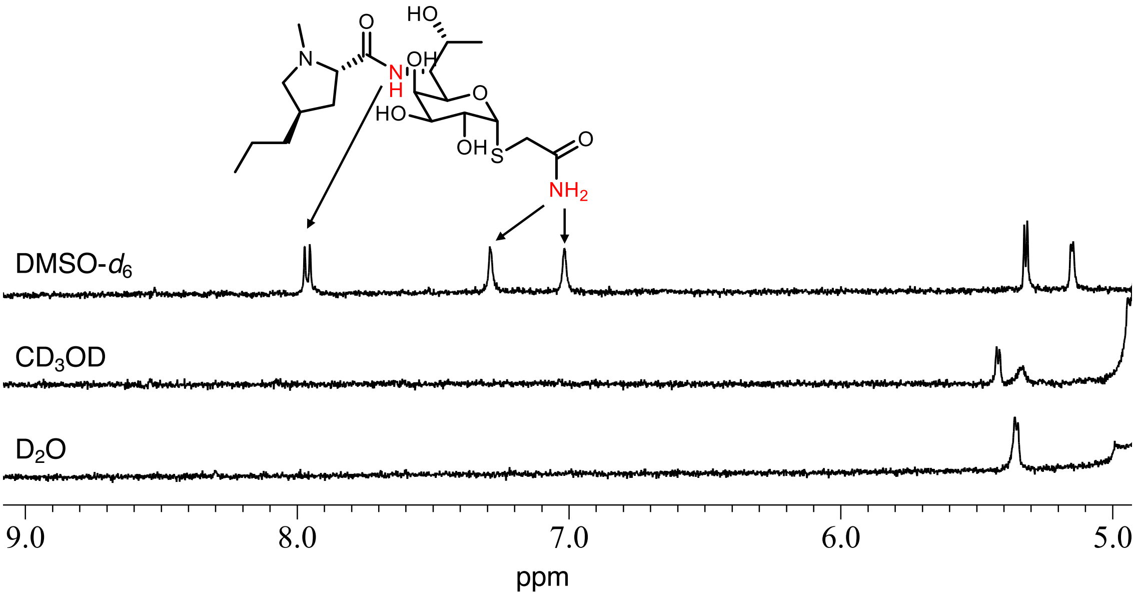

Supplementary Figure 10. Comparison of ^{1}H NMR spectra (amide moiety) among different solvents.

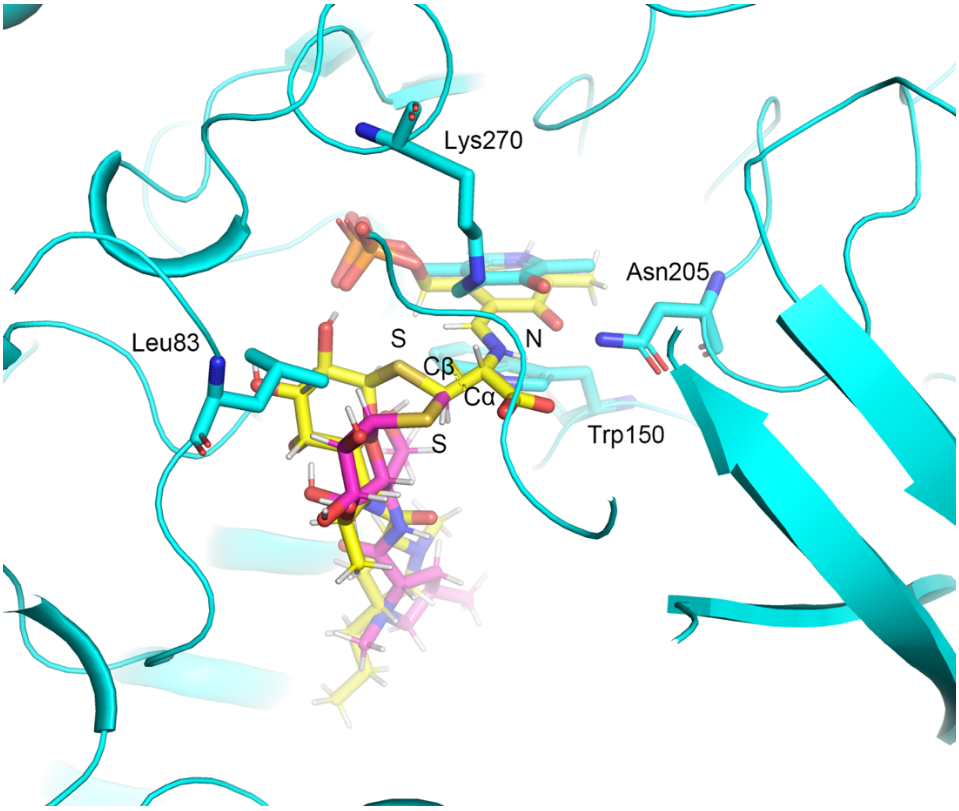

Supplementary Figure 11. Initial model of LmbF in complex with manually docked external aldimine models. Two possible conformations of docked external aldimine in the crystal structure of LmbF-PLP complex (cyan) are shown in yellow (the N-Cα-Cβ-S dihedral angle is -90°) and magenta (-180°).

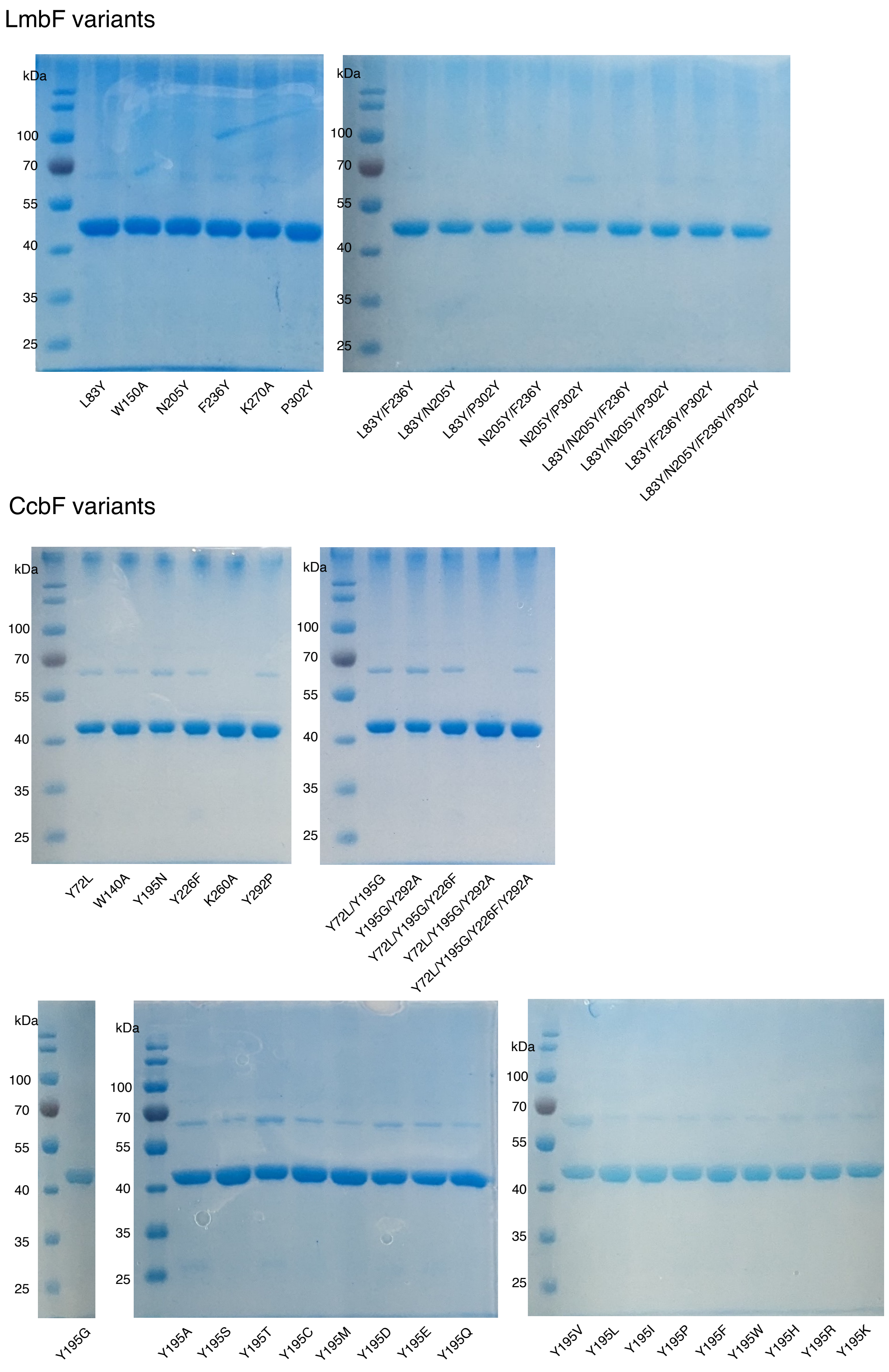

Supplementary Figure 12. **SDS-PAGE of purified enzymes.** These experiments were repeated independently three times with similar results.

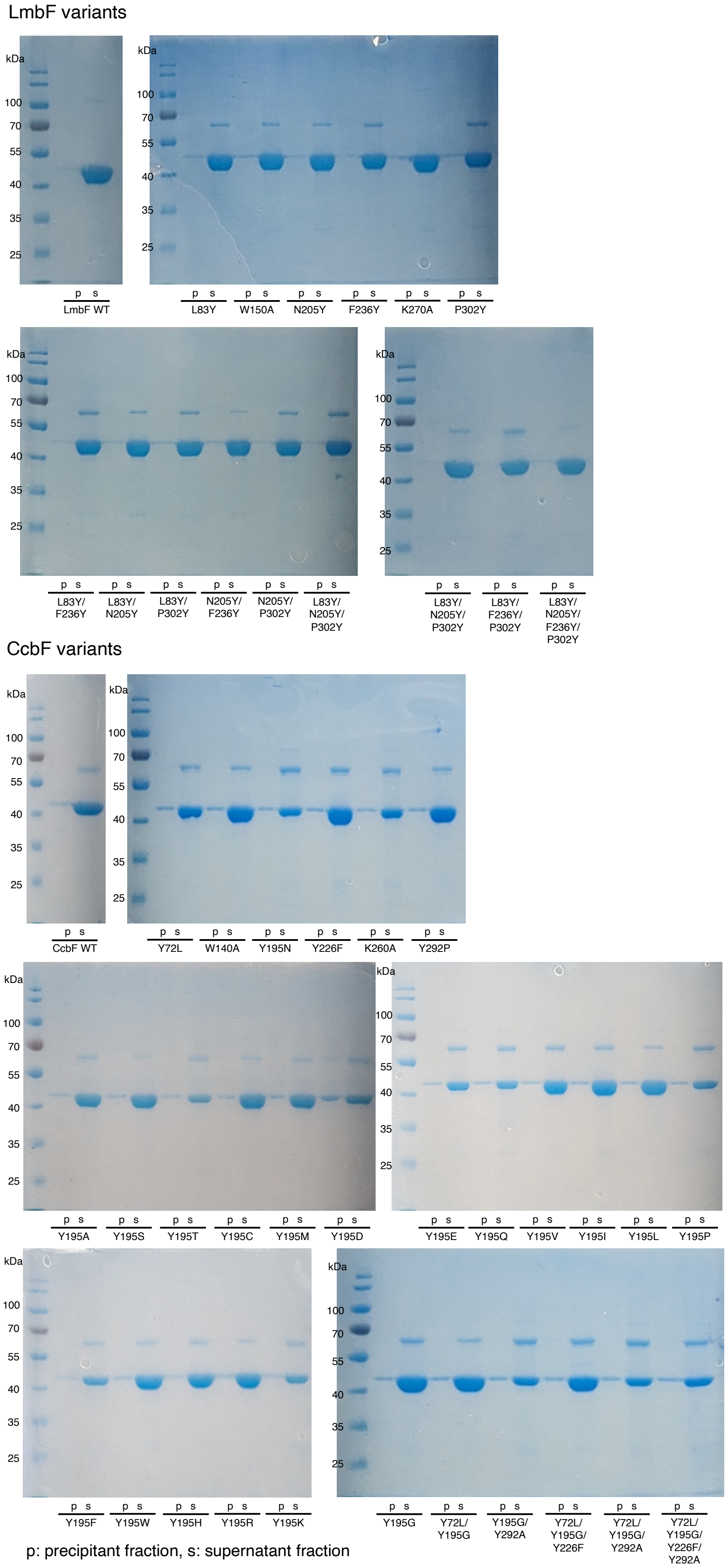

Supplementary Figure 13. Stability analysis of LmbF, CcbF and their mutants. These experiments were repeated independently three times with similar results.
