# Supplementary material for: Molecular basis for the diversification of lincosamide biosynthesis by pyridoxal phosphate-dependent enzymes
Source: Nat Chem. 2024 Dec 6;17(2):256–64. doi: 10.1038/s41557-024-01687-7 (PMC11794154; doi:10.1038/s41557-024-01687-7)
Supplement: Supplementary file 2 — Reporting Summary [file 41557_2024_1687_MOESM2_ESM.pdf]

nature portfolio

Corresponding author(s): Ikuro Abe

Last updated by author(s): Aug 13, 2024

Reporting Summary

Nature Portfolio wishes to improve the reproducibility of the work that we publish. This form provides structure for consistency and transparency in reporting. For further information on Nature Portfolio policies, see our Editorial Policies and the Editorial Policy Checklist.

Statistics

For all statistical analyses, confirm that the following items are present in the figure legend, table legend, main text, or Methods section.

n/a	Confirmed	
☐	☒	The exact sample size (*n*) for each experimental group/condition, given as a discrete number and unit of measurement
☐	☒	A statement on whether measurements were taken from distinct samples or whether the same sample was measured repeatedly
☒	☐	The statistical test(s) used AND whether they are one- or two-sided<br>*Only common tests should be described solely by name; describe more complex techniques in the Methods section.*
☒	☐	A description of all covariates tested
☐	☒	A description of any assumptions or corrections, such as tests of normality and adjustment for multiple comparisons
☐	☒	A full description of the statistical parameters including central tendency (e.g. means) or other basic estimates (e.g. regression coefficient) AND variation (e.g. standard deviation) or associated estimates of uncertainty (e.g. confidence intervals)
☒	☐	For null hypothesis testing, the test statistic (e.g. *F*, *t*, *r*) with confidence intervals, effect sizes, degrees of freedom and *P* value noted<br>*Give P values as exact values whenever suitable.*
☒	☐	For Bayesian analysis, information on the choice of priors and Markov chain Monte Carlo settings
☒	☐	For hierarchical and complex designs, identification of the appropriate level for tests and full reporting of outcomes
☒	☐	Estimates of effect sizes (e.g. Cohen's *d*, Pearson's *r*), indicating how they were calculated

Our web collection on statistics for biologists contains articles on many of the points above.

Software and code

Policy information about availability of computer code

Data collection	No software used
Data analysis	PHENIX-ver 1.19-4158, Phaser, AutoBuild, and phenix.refine in PHENIX-ver 1.19-4158, ccp4-7.1, XDS ver Feb 5 2021, Coot 0.9, PyMOL ver 2.0.6, AMBER 22, Gaussian 16 Rev. C.01., CPPTRAJ Version V6.18.1 (AmberTools), ZDOCK ver 3.0.2.

For manuscripts utilizing custom algorithms or software that are central to the research but not yet described in published literature, software must be made available to editors and reviewers. We strongly encourage code deposition in a community repository (e.g. GitHub). See the Nature Portfolio guidelines for submitting code & software for further information.

Data

Policy information about availability of data

All manuscripts must include a data availability statement. This statement should provide the following information, where applicable:

- Accession codes, unique identifiers, or web links for publicly available datasets
- A description of any restrictions on data availability
- For clinical datasets or third party data, please ensure that the statement adheres to our policy

The data supporting the findings of this study are available within the article and its Supplementary Information Files, source files. The topology and MD trajectory files used in this manuscript are freely available at Zenodo, https://doi.org/10.5281/zenodo.13309462. The crystallographic data that support the findings of this

study are available from the Protein Data Bank (http://www.rcsb.org). The coordinates and the structure factor amplitudes for the LmbF and CcbF were deposited under accession code 8KDL and 8KDK, respectively.

Human research participants

Policy information about studies involving human research participants and Sex and Gender in Research.

Reporting on sex and gender	n/a
Population characteristics	n/a
Recruitment	n/a
Ethics oversight	na/

Note that full information on the approval of the study protocol must also be provided in the manuscript.

Field-specific reporting

Please select the one below that is the best fit for your research. If you are not sure, read the appropriate sections before making your selection.

☒ Life sciences ☐ Behavioural & social sciences ☐ Ecological, evolutionary & environmental sciences

For a reference copy of the document with all sections, see nature.com/documents/nr-reporting-summary-flat.pdf

Life sciences study design

All studies must disclose on these points even when the disclosure is negative.

Sample size	For all statistical experiments, including kinetics analysis and conversion rate values were determined with n=3 as it is common practice in the field, exemplified in Nature, 527, 539-547 (2015) and J. Am. Chem. Soc. 141, 9964–9979 (2019).
Data exclusions	No data are excluded.
Replication	All attempts are performed independently and successfully replicated more than three times. We stated the number of replicates for each experiment in the paper. Furthermore, the reproducibility of the assays was confirmed by including appropriate positive and negative controls.
Randomization	This is not relevant to our study because, during the in vitro and structural analysis of the target enzymes, the products, catalyzed reactions, and enzyme structures were unknown, and none of the experiments involved assigning samples to test groups.
Blinding	Blinding is not relevant for this study because we use enzymatically synthesized substrates and mutant enzymes for in vitro reactions. During these analysis, the products, catalyzed reaction, and structure of enzymes were not known.

Reporting for specific materials, systems and methods

We require information from authors about some types of materials, experimental systems and methods used in many studies. Here, indicate whether each material, system or method listed is relevant to your study. If you are not sure if a list item applies to your research, read the appropriate section before selecting a response.

Materials & experimental systems

n/a	Involved in the study	
☒	☐	Antibodies
☒	☐	Eukaryotic cell lines
☒	☐	Palaeontology and archaeology
☒	☐	Animals and other organisms
☒	☐	Clinical data
☒	☐	Dual use research of concern

Methods

n/a	Involved in the study	
☒	☐	ChIP-seq
☒	☐	Flow cytometry
☒	☐	MRI-based neuroimaging
